# Activity and Heterogeneity of Astrocytes in Neurological Diseases: Molecular Mechanisms and Therapeutic Targets

**DOI:** 10.1002/mco2.70329

**Published:** 2025-08-22

**Authors:** Shijie Mao, Rui Qiao, Qi Wang, Ling Shen, Daxing Li, Xinchen Huo, Jindou Wang, Kunxuan Liu, Wenjing Chen, Tianhao Zhu, Beicheng Zhang, Shuo Leng, Ying Bai

**Affiliations:** ^1^ Department of Pharmacology School of Medicine Jiangsu Provincial Key Laboratory of Critical Care Medicine Southeast University Nanjing China; ^2^ State Key Laboratory of Oncology in South China Guangdong Provincial Clinical Research Center for Cancer Sun Yat‐sen University Cancer Center Guangzhou China; ^3^ Department of Radiology Center of Interventional Radiology and Vascular Surgery Zhongda Hospital Medical School Southeast University Nanjing China

**Keywords:** astrocytes, heterogeneity, neurological disorders, inflammation, neuron–astrocyte communication, biomarker

## Abstract

Astrocytes, the most prevalent glial cells in the central nervous system (CNS), play crucial roles in maintaining CNS homeostasis and responding to various pathological stimuli. They play key roles in neural development, neurotransmission, neuroinflammation, metabolic support, and tissue repair. Recent advancements in single‐cell sequencing have revealed the remarkable heterogeneity of astrocytes, with distinct subpopulations differentially contributing to disease progression in neurological disorders, including Alzheimer's disease, Parkinson's disease, Huntington's disease, amyotrophic lateral sclerosis, ischemic stroke, intracerebral hemorrhage, and multiple sclerosis. In addition, they play an important role in various behavioral neuropsychiatric disorders. This review highlights the dual roles of astrocytes in disease progression, driven by their diverse molecular profiles and functions. It outlines the key molecular mechanisms underlying astrocyte heterogeneity and their impact on neuroinflammation, neuronal support, and ionic balance regulation. Additionally, the review discusses potential therapeutic strategies targeting astrocytes to modulate these processes, aiming to improve treatment outcomes in neurological diseases. By elucidating the specific roles of astrocyte subsets in disease, this review seeks to advance the development of precision medicine for astrocyte‐related neurological disorders.

## Graphical abstract

Astrocytes display heterogeneity in various neurological disorders and behavioral neuropsychiatric disorders. Astrocytes influence the progression of neurological diseases through neuroinflammation, oxidative stress, metabolic abnormalities, intercellular interactions, and a spectrum of other pathophysiological processes. Research on astrocyte heterogeneity provides a reference for the development of subgroup‐specific therapies.

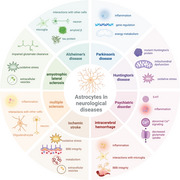

## 1 Introduction

Astrocytes, the most prevalent type of glial cells, are extensively distributed throughout the central nervous system (CNS) [1, 2]. Astrocytes were thought to be divided into two subtypes in the past. However, with the continuous development of sequencing technology, astrocytes are gradually divided into more subtypes according to different assays. Each subtype has its own specific molecular markers to distinguish it from the others [3, 4]. Especially with the rise of single‐cell sequencing, analysis of its results has revealed differences in gene expression between different subsets of astrocytes, which may reflect their different roles and functions in the nervous system. At the same time, it can also help us better understand the respective roles and mechanisms of different subgroups in the pathological state of related diseases, such as neurotransmitter uptake, energy metabolism, ionic balance, and immune regulation abnormalities, and provide new treatment strategies for diseases [5].

The distribution of astrocytes in the brain is widespread and heterogeneous. Their distribution density and morphological differences in different brain regions provide an important guarantee for the normal function of the nervous system [1]. Astrocytes have complex morphology and structure, which leads to many different functions in the brain. The blood–brain barrier (BBB) consists of tightly joined endothelial cells that help regulate brain homeostasis and shield neurons from harm. When this barrier is compromised, immune cells and plasma proteins can infiltrate the brain tissue, potentially triggering neuroinflammatory responses [6]. They perform a range of functions, such as the maintenance of the integrity of BBB, neuronal signal communication participation, neurotransmitter metabolism regulation, the provision of nutrition and support, and the removal of neuronal waste [7, 8, 9, 10]. Endo et al. [1] discovered that hundreds of genes were abundant in astrocytes and shared among astrocytes through gene knockout. They demonstrated that their functions were related to metabolism, cholesterol, neurotransmitter absorption, and biosynthesis [1]. It can be found that the morphological and functional changes of astrocytes are inseparable from the occurrence and development of CNS diseases. In 2021, more than 3 billion people worldwide suffered from neurological disorders. Neurological degenerative diseases rank among the primary causes of mortality and disability [10].

In addition, although mental disorders are not classified as neurological diseases, with the gradual deepening of research on mental disorders, more and more signaling pathways and molecular markers affecting the development of mental disorders have been discovered. This review will elucidate the mechanism of action of astrocytes in a variety of CNS diseases and the target molecules that may be involved in psychiatric disorders. The vast majority of the articles we select are from the past 5 years and have a high impact factor (>8.5). If there are fewer high‐score articles related to a certain disease, we will appropriately lower the score standard and screen for higher‐quality articles.

## 2 Astrocytes in Neurodegenerative Diseases

**FIGURE 1:**
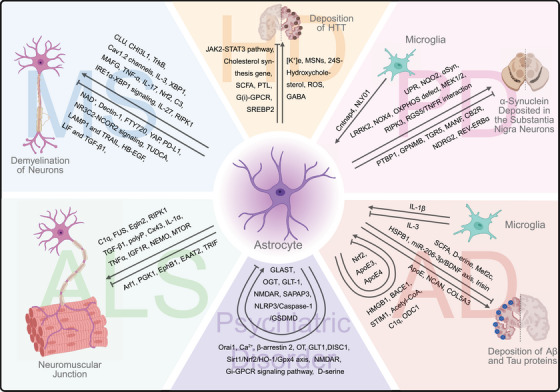
Interactions of astrocytes with neurons and other glial cells in multiple sclerosis, amyotrophic lateral sclerosis, Huntington's disease, Parkinson's disease, Alzheimer's disease, and psychiatric disorders. The six different regions describe the key pathways or molecules involved in the six diseases in which astrocytes act on themselves or with neurons or microglia. Astrocytes improve or worsen disease symptoms through these actions. The figure was created using BioRender.com.

Astrocytes, the predominant glial cells in the CNS, play a critical role in maintaining homeostasis and progression of neurological diseases [11], including multiple sclerosis (MS), Alzheimer's disease (AD), Parkinson's disease (PD), Huntington's disease (HD), and amyotrophic lateral sclerosis (ALS) [12]. Recent research has highlighted the potential involvement of astrocytes in the development and progression of neurological diseases (Figure 1). This review delves into the current understanding of general mechanisms of astrocyte regulation in several types of neurological diseases and their potential as therapeutic targets.

### 2.1 Alzheimer's Disease

AD is the leading cause of dementia [13] and ranks among the most prevalent neurodegenerative diseases [12]. According to the ATN (A: amyloid, T: phosphorylated tau, N: neurodegeneration) framework constructed by Jack and colleagues [14], the biomarkers amyloid β (Aβ) and phosphorylated tau are fundamental to the diagnosis of AD. The initial phase of AD occurs in parallel with the accumulation of Aβ, which further induces the occurrence and development of tau pathology [13, 15]. Guo et al. [16] measured the plasma levels of Aβ42/Aβ40, p‐Tau181, and glial fibrillary acidic protein (GFAP, a structural protein mainly present in astrocytes) and set thresholds, which served as a significant reference for the early diagnosis of AD in China. These findings highlight that Aβ and tau are crucial in AD research. There is a strong link between gliosis and AD. Proliferative glial cells mainly surround Aβ plaques and tau neurofibrillary tangles. Astrocytes in AD exhibit a dual role, contributing to either the excessive accumulation or clearance of Aβ plaques and tau aggregates.

Based on current single‐cell sequencing results, changes in transcriptional levels of astrocytes in AD patients correlate with specific case indicators. Cho et al. [17] demonstrated that differentially expressed genes (DEGs) were enriched in astrocytes after AD occurred. Among them, the expression level of *CLIC4* was in high agreement with the final diagnosis of AD. The similarities and differences in the transcription of astrocytes in different brain regions reveal the heterogeneity of astrocytes at the gene expression and functional levels on the basis of commonality, which explains why certain brain regions are more susceptible after the occurrence of AD. For example, M9, a module widely expressed by astrocytes, is associated with the assembly of cell junctions. Another example is the subtype‐specific and region‐specific modules associated with astrocytes: M19 in the thalamus, which is associated with acoustic hedgehog signaling; M12 related to preneuronal development in the brain and M7 associated with synaptic membranes. There is also the expression of specific modules of astrocytes connected with AD: the expression of module M28, which is associated with metal ion homeostasis, overlaps with the expression of APOE^+^ (M0) and reactive (M3) astrocytes [18]. The different subtypes of astrocytes derived from sequencing in more detail may have synergistic or antagonistic functions with each other. Alexandra Grubman identified multiple astrocyte subtypes a1, a2, a3, a4, a5, a6, a7, and a8 in the entorhinal cortex (EC) of AD and control individuals using single‐nucleated RNA sequencing technology. Each subtype has its own unique marker genes and functional characteristics. Among them, a1 subtype astrocytes are rich in genes related to ribosome, mitochondria, neuronal differentiation, and heat shock response, which may fight cell stress and cell damage. The a2 subtype, on the other hand, exhibits downregulation of these processes and is associated with the neuroinflammatory and pathological processes of AD [19]. Griswold et al. [20] identified the astrocyte subset Cluster 20 by single‐nucleated RNA sequencing technology. Among them, *IFITM3*, *OLFM1*, *B2M*, *TAPBP*, and *CHI3L1* genes were highly expressed in Cluster 20. These gene expression products are also markers of type A1 reactive astrocytes (RAs), which suggests that the Cluster 20 subset may be involved in neuroinflammatory processes in AD [20]. Notably, the heterogeneity of astrocytes may also correlate with age. After lipopolysaccharide (LPS) treatment, astrocytes exhibit higher heterogeneity upon activation, particularly in the vicinity of the corpus callosum, leptomeninges, and ventricles [21]. The distinct functions of astrocytes in AD progression are described in detail below, particularly their interactions with neurons and microglia, as well as their own regulatory roles (Table 1).

**TABLE 1:** Molecules and mechanisms associated with astrocytes in Alzheimer's disease.

	Key molecules	Implications	References
Astrocyte–neuron	SCFAs	Upregulate GS in astrocytes, enhance glutamate–glutamine shuttling, reduce Aβ deposition and tau hyperphosphorylation	[22]
	APOE	In neurons, APP transported by APOE to lipid rafts interacts with APOE4 to produce more Aβ; astrocytes reduce the clearance of Aβ due to disorders in cholesterol metabolism.	[23]
	D‐Serine	Impaired glycolysis in astrocytes reduces l‐/d‐serine synthesis and decreases the binding rate of d‐serine to NMDAR.	[24]
	*NCAN*, *COL5A3*	Upregulation of gene expression encoding *NCAN* and *COL5A3* in Astro0 and Astro1 leads to glial scarring and inhibition of axonal regeneration.	[25]
	Mef2c	Tau hyperphosphorylation inhibits Mef2c and decreases the content of miR‐133a‐3p, increases the expression of A1R to induce the upregulation of lipocalin 2 and promote the activation of astrocytes and inflammatory response.	[26]
	HSPB1	HSPB1 secreted by reactive astrocytes surrounding amyloid plaques is taken up by astrocytes and neurons, leading to reduced astrocytic inflammation and pathological tau levels.	[27]
	miR‐206‐3p/BDNF axis	aFGF stimulates astrocytes to produce AEVs_‐Aβ+H_ and inhibits AEP activation through the miR‐206‐3p/BDNF axis to reduce Aβ burden.	[28]
Astrocyte–microglia		The accumulation of microglia surrounding Aβ plaques in the hippocampus promotes neurotoxic astrocyte activation induces GABAergic signaling and reduces glutamatergic signaling.	[29]
	IL‐1β	NLRP3 inflammasomes assembled around Aβ activate microglia and astrocytes to overrespond to IL‐1β.	[30]
		Coculture of astrocytes and microglia can significantly reduce the deposition of intracellular α‐SYN and Aβ than astrocytes alone.	[31]
	IL‐3	Increased expression of IL‐3Rα in microglia enhances astrocyte responses to aberrant and persistent expression of IL‐3 and enhances aggregation and clearance of Aβ and tau protein aggregates.	[32]
Astrocyte autoregulation	APOE4	APOE4 promotes the secretion of GPC‐4 by neurotoxic astrocytes through the NF‐κB pathway. The binding of GPC‐4 to APOE4 greatly affects the distribution of LRP1 on the cell membrane and increases molecular transport, resulting in over uptake and phosphorylation of tau protein.	[33]
	APOE3, APOE4	Tamoxifen reduces the deposition of Aβ plaques and reduces plaque compactness by selectively decreasing the content of APOE3 and APOE4 in astrocytes.	[34]
	APOE4	Decreasing APOE4 levels in astrocytes results in the transfer of Aβ deposition from the brain parenchyma to cerebral blood vessels, reducing Aβ‐mediated glial hyperplasia.	[35]
	Nrf2	Enhancement of Nrf2 expression attenuates the recruitment of the NF‐κB subunit p65, thereby decreasing the induction of reactive astrocytes. Activation of Nrf2 also inhibits type I interferons and antigen presentation pathways.	[36, 37]
	HMGB1	Direct exposure to TauO results in HMGB1 release, which promotes SASP and leads to tau protein deposition. EP and GA can inhibit this pathway and improve neurological function.	[38]
	SQSTM1	Aβ transiently induces *LC3B* gene expression and prolongs transcription of SQSTM1. Aβ‐induced astrocyte autophagy accelerates the urea cycle and aliphatin degradation pathways.	[39]
	BACE1	Deletion of BACE1 reduces cleavage of the IRβ subunit, and phosphorylation of signaling molecules such as P38, ERK1/2, and cJun upregulates the expression of *Clu* and *Cxcl14* to enhance Aβ clearance.	[40]
	STIM1	Increased STIM1 expression due to early plaque deposition results in a decrease in ER Ca^2+^ levels and a decrease in Ca^2+^ signaling.	[41]
	Acetyl‐CoA	Excess FA load surpassing astrocyte OXPHOS capacity elevates acetyl‐CoA, triggering astrocyte reactivity through enhanced STAT3 acetylation.	[42]
	C1q	Deletion of C1q inhibits CCPs, thereby reducing astrocyte–synaptic association and reducing astrocyte phagocytosis of excitatory synapses in TauP301S mice.	[43]
	ODC1	The presence of Aβ changes the urea cycle from linear to cyclical: the reduction of ODC1 promotes the conversion of ornithine to dextran, eliminating dextran, ammonia, and H2O2 to reduce GABA and alleviate memory impairment.	[44]
Peripheral immunity		Tregs promote A2‐like phenotypic differentiation by inhibiting C3‐positive astrocytes.	[45]
Exercise	Irisin	Exercise‐induced irisin acts on αV/β5 integrins in astrocytes, downregulating the ERK–STAT3 signaling pathway and increasing the release of NEP from astrocytes, thereby degrading more Aβ.	[46]
Circadian rhythm	*Chi3l1*	Deletion of BMAL1 or CLOCK/NPAS2 inhibits *Chi3l1* (circadian‐regulated) expression, reducing YKL‐40 levels and enhancing astrocyte phagocytosis of zymosan/Aβ.	[47]

Abbreviations: A1R, adenosine receptor 1; acetyl‐CoA, acetyl coenzyme A; AEP, asparagine endopeptidase or δ‐secretase; AEVs_‐Aβ+H_, astrocyte‐derived extracellular vesicles stimulated by aFGF; APOE, lipoprotein E; APP, amyloid precursor protein; BACE1, beta‐site amyloid precursor protein cleaving enzyme 1; BDNF, brain‐derived neurotrophic factor; BMAL1, brain and muscle ARNT‐like protein 1; C1q, complement component 1q; C3, complement component 3; CCPs: complement classical pathway; *Chi3l1*, chitinase 3‐like protein 1; *COL5A3*, collagen type V alpha 3 chain gene; EP, ethyl pyruvate; ER, endoplasmic reticulum; ERK, extracellular signal‐regulated kinase; ERK1/2, extracellular signal‐regulated kinase 1 and 2; FA, fatty acid; GA, glycyrrhizic acid; GABA, gamma‐aminobutyric acid; GPC‐4, glypican‐4; GS, glutathione synthetase; HMGB1, high mobility group box 1; HSPB1, heat shock protein β1; IL‐1β, interleukin‐1β; IL‐3, interleukin‐3; IL‐3Rα, CD123 or receptor for IL‐3; IRβ, insulin receptor β subunit; LRP1, low‐density lipoprotein receptor‐related protein 1; Mef2c: myocyte enhancer factor 2C; *NCAN*, neurocan gene; NEP, neprilysin; NMDA, N‐methyl‐d‐aspartate; NMDAR, NMDA receptors; NPAS2, neuronal pas domain protein 2; Nrf2, NF‐E2‐related factor 2; ODC1, ornithine decarboxylase 1; OXPHOS, oxidative phosphorylation; SASP, senescence‐associated secretory phenotype; SCFAs, short‐chain fatty acids; SQSTM1, sequestosome1; STAT3, signal transducer and activator of transcription 3; STIM1, Ca^2+^ sensor; TauO, tau oligomers; Tregs, regulatory T cells; α‐SYN, alpha‐synuclein.

#### 2.1.1 Interactions Between Astrocytes and Neurons

Astrocytes are closely related to neurons, both structurally and functionally. However, due to excessive deposition of Aβ or tau, the connection between astrocytes and neurons is altered, affecting the interaction between astrocytes and neurons. Barbar et al. [48] discovered that the *ITGA6*‐encoded CD49f was a novel marker for astrocytes. They found that the CD49f^+^ cell population was rich in mature astrocytes, while CD49f^−^ cells contained transitional astrocytes and immature astrocytes by single‐cell sequencing. There is a difference in the response of the two to inflammatory stimuli. CD49f^+^ astrocytes are able to support the electrophysiological properties and synapse formation of neurons, promoting neuronal maturation and connection [48]. Luo et al. [49] analyzed five subregions of the hippocampus–EC system and found that the connections between astrocytes and neurons were greatly altered in the EC of AD patients. PSAP induces the transformation of astrocytes to the A1 phenotype, resulting in the proliferation of A1 astrocytes [49]. Abnormalities in neurons cause abnormal activation of astrocytes. Zhou et al. [26] found that hyperphosphorylation of neuronal tau protein could inhibit the transcription factor Mef2c. The reduction of Mef2c could inhibit the transcription of miR‐133a‐3p, further increasing the expression of A1R. This ultimately induced upregulation of lipocalin 2 (LCN2) and promoted the activation and inflammatory response of astrocytes [26]. The action of astrocytes can delay the progression of AD and slow down neuronal function damage in some ways. Sun et al. [22] found that excessive short‐chain fatty acids (SCFAs) mainly acted on astrocytes and increased glutamine synthetase expression, thereby enhancing glutamate–glutamine shuttle between astrocytes and neurons. Finally, it promotes the synthesis of glutathione in neurons, efficiently decreasing the deposition of Aβ and the hyperphosphorylation of tau, further alleviating oxidative damage and protecting neurons [22]. Yang et al. [27] found increased levels of heat shock protein β1 (HSPB1) secreted by RAs clustered around Aβ plaques. HSPB1 was taken up by astrocytes or neurons, attenuating the inflammatory response caused by RAs and reducing the aggregate content of phosphorylated tau [27]. Peng and colleagues [28] found that acidic fibroblast growth factor (aFGF) stimulated the production of AEVs_‐Aβ+H_ (astrocyte‐derived extracellular vesicles [ADEVs] stimulated by aFGF) in astrocytes and inhibited the activation of δ‐secretase (AEP) through the miR‐206‐3p/BDNF axis to reduce Aβ burden.

Unfortunately, astrocytes can also cause some damage to neurons during the AD process, increasing the disease burden. Damage to astrocytes leads to neurodegeneration, synaptic retraction, and ultimately cognitive deficits [50]. Past studies have identified many AD‐related risk genes expressed by astrocytes, such as *APOE*, *SLC1A2*, *CLU*, *MEF2C*, *IQCK*, and so on [1, 51, 52]. At present, the research focuses more on lipoprotein E (APOE). In stressful states, APOE is predominantly produced by astrocytes in the CNS. Li et al. [23] found that astrocytes transported amyloid precursor protein (APP) from neurons to neurons’ cholesterol‐rich lipid rafts via APOE, and APOE4 in lipid rafts induced more cholesterol accumulation. Eventually, this led to an increase in APP, which caused neurons to produce more Aβ. At the same time, they also found that due to the disorder of cholesterol metabolism in the brain, astrocytes were unable to use cholesterol normally, thus reducing the clearance of Aβ [23]. In turn, APOE stimulated the polarization of astrocytes. Griswold et al. [20] found that high levels of APOE expression might stimulate the formation of type A1 RAs. Of course, neurons can also express a certain amount of APOE. Blumenfeld et al. [53] found that APOE4 represented the most significant genetic risk factor for late‐onset AD. Neuronal APOE4 could elicit glial cell responses and eventually led to neurodegeneration [53]. In addition to lipid metabolism, glucose metabolism in astrocytes is also greatly affected. Johnson and his team [54] found that glucose metabolism‐related proteins were significantly elevated in the early stages of AD. Le Douce et al. [24] found that in the early stages of AD, the glycolysis of star gum was impaired, resulting in a decrease in l‐/d‐serine synthesis. This led to a decrease in the binding rate of D‐dserine to NMDA receptors (NMDAR), which impaired synaptic plasticity and memory, and ultimately led to impaired cognitive function [24]. Zhou et al. [25] reported elevated expression of *NCAN* and *COL5A3* genes in Astro0 and Astro1, which might hinder axon regeneration by promoting glial scarring.

#### 2.1.2 Interaction Between Astrocytes and Microglia

In the nervous system, there is a strong connection between astrocytes and microglia. Astrocytes and microglia interact with each other in terms of morphology and function. Mallach et al. [29] found that microglia clustered around hippocampal Aβ plaques, which promoted the differentiation of astrocytes into neurotoxic phenotypes. Neurotoxic astrocytes significantly influence hippocampal neurons by enhancing GABAergic signaling and suppressing glutamatergic signaling, disrupting synaptic balance [29]. Rostami et al. [31] found that the coculture of astrocytes and microglia led to a notable decrease in intracellular deposition of α‐SYN and Aβ compared with astrocytes alone. Microglia are important immune cells of the CNS, and their crosstalk with astrocytes is becoming increasingly important in AD. Singh [55] noted that aberrant activation of glial cells might mediate neuroinflammation, while chronic or uncontrolled inflammatory responses might lead to AD. Signaling and interaction between astrocytes and microglia can be achieved through certain inflammatory mediators. McAlpine et al. [32] proved that when AD occurred, astrocytes continuously produced interleukin‐3 (IL‐3). Microglia would increase the expression of IL‐3Rα after recognizing Aβ deposits, strengthened the response to IL‐3, and ultimately enhanced the ability to aggregate and clear Aβ and tau protein aggregates [32]. Lopez‐Rodriguez and colleagues [30] found that the presence of NLRP3 inflammasomes around Aβ plaques could activate microglia and promote microglia response to subsequent excessive IL‐1β stimulation by LPS. Thus, astrocytes were activated and produced a strong chemotaxis response to IL‐1β within the hippocampus [30].

#### 2.1.3 The Self‐Regulatory Effects of Astrocytes

In addition to interacting with other nerve cells, astrocytes themselves also have a variety of mechanisms that affect the AD process, accelerating or slowing down the deposition of Aβ and tau. Saroja and colleagues [33] demonstrated that the neurotoxic astrogum secreted GPC‐4 in the presence of APOE4 via the NF‐κB pathway. GPC‐4 bound to APOE4 and greatly affected the distribution of LRP1 on the cell membrane and increased transport, resulting in over uptake and phosphorylation of tau protein. Xiong et al. [35] proved that decreasing APOE4 levels in astrocytes resulted in the transfer of Aβ deposition from the brain parenchyma to cerebral blood vessels. Although CAA was more prevalent, Aβ‐mediated glial hyperplasia was reduced [35]. Mahan's laboratory [34] found that tamoxifen selectively reduced the levels of APOE3 and APOE4 in astrocytes, which substantially reduced the deposition of Aβ plaques while reducing plaque compactness, resulting in a significant reduction in the total amount of cortical Aβ‐associated neurodystrophy. Mi et al. [42] found that acetyl CoA levels increased when the FA load exceeded the star gum oxidative phosphorylation (OXPHOS) capacity, inducing astrocyte reactivity by enhancing STAT3 acetylation. Besides studies on fat metabolism, Gaikwad et al. [38] demonstrated that direct exposure to TauO led to HMGB1 release, promoted SASP, and led to tau protein deposition. EP and GA could reduce TauO and senescent cell burden, inhibit HMGB1 release, and improve neurological function [38]. Regulation of the structure and function of astrocytes can be achieved by influencing gene expression. It was found that the enhancement of the expression of the transcription factor Nrf2 attenuated the p65 recruitment of the NF‐κB subunit, thereby reducing the induction of RAs. Activation of Nrf2 also mitigated the progression of AD by inhibiting type I interferons and antigen presentation pathways [36, 37]. Kim's research team [39] found that Aβ could transiently induce the gene expression of LC3B and prolong the transcription of the SQSTM1 gene, promoting autophagy in astrocytes. Aβ‐induced astrocyte autophagy could accelerate the urea cycle and fatty acid degradation pathway [39]. Ju et al. [44] found that in the presence of Aβ, the urea cycle changes from linear to cyclical: ODC1 is reduced, which promoted the conversion of ornithine to dextran, and eliminated dextran, ammonia, and H_2_O_2_ to reduce GABA and alleviate memory impairment. In addition to the above, the modification of molecules in astrocytes is also strongly linked to AD. Zhou et al. [40] found that BACE1 deletion reduced the cleavage of the β subunit of IR, enhanced phosphorylation of signaling molecules such as P38, ERK1/2, and cJun, and upregulated the expression of *Clu* and *Cxcl14*, thereby enhancing Aβ clearance. Ca^2+^ is an important mediator of astrocyte signaling. Lia et al. [41] suggested that at the beginning of plaque deposition, the expression of the Ca^2+^ sensor STIM1 in the astrocytes of AD female mice declined and the concentration of Ca^2+^ in the endoplasmic reticulum decreased, which caused a sharp drop in Ca^2+^ signaling, resulting in a lessening in the activity and synaptic plasticity of the astrocytes. Finally, in terms of immunity, the role of astrocytes also has a certain impact. Dejanovic et al. [43] found that the absence of complement C1q could inhibit the classical complement pathway, which reduced astrocyte–synaptic association in TauP301S mice, thereby reducing astrocyte phagocytosis of excitatory synapses.

#### 2.1.4 Other Mechanisms

In addition to the above‐mentioned aspects, there are many other factors that can affect the AD process. Stym‐Popper and colleagues [45] found that peripheral immune cell Tregs favored activation to the A2‐like phenotype by inhibiting C3‐positive astrocytes, thereby regulating the balance of RA subtypes in Aβ pathology. Studies by Kim et al. [46] showed that exercise slowed the progression of AD. Exercise could produce irisin, which acted on αV/β5 (the receptor of irisin), thereby downregulating the conduction of the ERK–STAT3 signaling pathway, resulting in increased NEP release and more degradation of Aβ [46]. Lananna et al. [47] found that circadian rhythm could modulate *Chi3l1*, and the deletion of core clock proteins—BMAL1 or Clock/NPAS2 inhibited the expression of *Chi3l1*, thereby reducing the content of YKL‐40 and leading to enhanced phagocytosis of zymosan particles and Aβ by astrocytes.

### 2.2 Parkinson's Disease

PD holds the second position among the prevalent neurodegenerative ailments, distinguished by the depletion of dopamine neurons in the compact area of the substantia nigra and the abnormal accumulation of misfolded α‐synuclein within Lewy bodies [56]. Its etiology is still unknown and therefore treatment strategies are limited. Current research has found that the origin and causation of PD are intricate and involve multiple factors, associated with genetic, environmental, and aging factors [57]. Astrocytes represent the most preponderant glial cells within the CNS and play multiple physiological roles, including secreting neurotrophic molecules, regulating synaptic transmission, maintaining water and ion homeostasis, and modulating BBB permeability. Astrocytes exert an influence on PD through multiple mechanisms, including neuroinflammation, energy metabolism, gene regulation, and signaling (Table 2). Studies have shown that both dysfunctional astrocytes and RA proliferation contribute to the pathogenesis and advancement of PD [58, 59].

**TABLE 2:** Molecules and mechanisms associated with astrocytes in Parkinson's disease.

Key molecules	Implications	References
NLY 01	Blocks microglia‐induced A1 astrocyte transformation	[63]
UPR	Neurodegeneration/apoptosis or leads to neurological dysfunction	[64]
NQO2	Plays a toxic role in dopaminergic degeneration and neuroprotection	[65]
α‐Syn	Impairs essential cellular processes	[66]
RNA‐binding protein PTBP1	Conversion of specific glial cell subpopulations to neurons due to deficiency	[67]
GPNMB	Inhibits astrocyte‐mediated neuroinflammation	[68]
TGR5	Alleviates the advancement of PD	[69]
LRRK2	The G2019S mutation of LRRK2 has been distinguished as a causative mutation in PD.	[70]
MANF	Protects and repairs dopaminergic neurons	[71]
CB2R	Mitigates nlrp3‐triggered neuroinflammation and improves the disorder manifestations related to PD	[72]
NDRG2	Rectifies astrocyte malfunction and opposes the development of particular neurological disorders	[73]
NOX4	Induces mitochondrial dysfunction in hippocampal astrocytes	[74]
OXPHOS	Impaired neuronal support function due to OXPHOS defects	[75]
MEK1/2 signaling	Suppression of the MEK1/2 signaling pathway can diminish the inflammatory traits exhibited by mutant astrocytes and restore the formation of the BBB.	[76]
REV–ERBα	Attenuates astrocyte activation and reduces dopaminergic neuron damage	[77]
Cntnap4	Modulate the communication between glial cells	[78]
RIPK3 signaling pathway	Neuronal DAMPs intensify the process of neurodegeneration by means of astrocytic RIPK3 signaling.	[79]
RGS5/TNFR interaction	Activates astrocytes and causes chronic neuroinflammation.	[80]

Abbreviations: CB2R, cannabinoid receptor type 2; DAMPs, damage‐associated molecular patterns; GPNMB, glycoprotein nonmetastatic melanoma protein B; MANF, midbrain astrocyte‐derived neurotrophic factor; NQO2, quinone oxidoreductase 2; OXPHOS, oxidative phosphorylation;PD, Parkinson's disease; TGR5, takeda G protein‐coupled receptor 5; UPR, unfolded protein response.

Using single‐cell sequencing technology, researchers have made a series of discoveries in the field of astrocyte heterogeneity in PD. Smajić’s team [60] performed single‐cell sequencing analysis of brain tissues from PD patients, and identified five subpopulations of astrocytes characterized by differences in the expression of the following genes: ELMO1, ADGRV1, VAV3, LRRC4C, and CD44, which were specifically highly expressed [60]. In the PD rat model, Wu's team [61] used single‐cell RNA sequencing to analyze the heterogeneity of astrocytes in the substantia nigra region and identified five subtypes (astrocyte‐0 to astrocyte‐4). The subtype‐specific marker genes were as follows: astrocyte‐0 exhibited expression of *Plcb1* and *Trpm3*; astrocyte‐1 was marked by the presence of *Snhg11* and *Syt*1; astrocyte‐2 displayed the characteristic expression of *Pex5l* and *Tmeff2*; astrocyte‐3 showed the expression of *Vim* and *Nckap5*; and astrocyte‐4 was distinguished by the presence of *Gfap* and *Map7*. Metabolic analysis showed that astrocyte subtypes in the PD model exhibited significant metabolic pathway alterations, including: abnormal energy metabolic pathways in astrocyte‐0, astrocyte‐1, and astrocyte‐2 subtypes; involvement of astrocyte‐0 subtypes in phospholipid metabolism; and a significant increase in amino acid metabolic pathway activity in astrocyte‐4 subtypes [61]. Studies on the comorbidity mechanism of PD and depression (MDD) have found that the Astro_3 isoform among the eight astrocyte isoforms exhibits the most significant transcriptomic changes in the comorbid state. The Astro_3 subtype not only highly expresses the risk genes related to PD–MDD comorbidities, but also mainly participates in the regulation of chemical synaptic function, neural projection development and energy metabolism [62]. In conclusion, the development of single‐cell sequencing technology has revealed the molecular features and potential mechanisms of PD progression, providing evidence for further exploration in the future.

#### 2.2.1 Heterogeneity of Astrocytes in PD

Abnormal function of astrocytes has a bifunctional role in PD. On the one hand, astrocytes exacerbate neurodegenerative damage in PD through inflammatory responses and neurotoxic phenotypes (A1 astrocytes); on the other hand, astrocytes directly influence neuronal survival and function in PD by modulating pathways, among them neuroinflammation and oxidative stress. Glucagon‐like peptide‐1 receptor (GLP1R) agonists can serve as prospective influences for treating and protecting against neurological disorders like AD and PD. NLY01, which is a powerful GLP1R agonist, exerts a neuroprotective effect by directly suppressing the transformation of astrocytes to the A1 neurotoxic phenotype mediated by microglia [63]. GPNMB, functioning as a transmembrane glycoprotein, also acts as a receptor manifested on astrocytes. The molecule CD44 has the capacity to couple with GPNMB. It is hypothesized that this binding interaction could potentially confer neuroprotective benefits by curtailing the neuroinflammation mediated by astrocytes, with the process relying on CD44 in a crucial way [68]. CB2R, which belongs to the family of GPCR, presents itself as a prospective candidate for therapy of inflammation‐linked disorders. When activated on astrocytes, CB2R can efficiently mitigate nlrp3‐triggered neuroinflammation and improve the pathological features associated with PD in mice [72]. In contrast to the protective mechanisms described above, the receptor‐interacting protein kinase‐3 (RIPK3) signaling pathway has been described as a crucial modulator of neuroinflammation. Chang et al. [79] found that factors released from dead neurons induced astrocytic RIPK3 signaling via late glycosylation end product receptor signaling, which conferred functional activity in inflammation and neurotoxicity. In addition, Yin et al. [80] found that adjuster of G protein signaling pathway 5 (RGS5), a key regulator of the TNF signaling pathway, can activate astrocytes leading to chronic neuroinflammation. Blocking astrocyte RGS5/TNFR interactions may be a promising treatment scheme for neuroinflammation‐associated neurodegenerative diseases [80].

#### 2.2.2 Important Genes and Proteins in PD

Researchers have found that a variety of proteins play important roles in PD. As mentioned above, aberrant aggregation of α‐synuclein is one of the important features of PD, and the aggregation of αSyn disrupts important cellular functions such as synaptic function, mitochondrial integrity, and protease inhibitors, which ultimately leads to neuronal cell death [56]. In addition to this, quinone oxidoreductase 2 negatively regulates astrocyte autophagy and neuroprotection, thus playing a toxic role in dopaminergic degeneration; depletion of a single RNA‐binding protein, PTBP1, converts astrocytes into neurons thus reversing PD, and ursodeoxyn powder exerts a role in attenuating PD through inhibition of astrocyte‐mediated inflammation by the TGR5 [65, 66, 67, 69]. Stress triggered by the endoplasmic reticulum unfolded protein response (UPR) in PD leads to degeneration and death of dopamine neurons. Midbrain astrocyte‐derived neurotrophic factor safeguards and restores dopamine neurons through modulating UPR, which holds the potentiality to work as a new aim for PD treatment [64, 71]. The development of PD is closely associated with mutations in several genes, and the *G2019S* mutation in *LRRK2* was identified to be pathogenic, underscoring the promotion of PD by gene mutations [70]. In recent years, gene editing and cell reprogramming technologies have provided novel ideas for the treatment of PD: using technologies such as the CRISPR system and adeno‐associated virus (AAV) vectors, astrocytes can be transformed into GABAergic neurons to replace neural circuit deficits as a result of the deterioration of dopamine neurons [81]. In addition, the combined application of transcription factors and miRNAs has shown the potential to reprogram astrocytes to rebuild neural networks [82]. In terms of regulation of astrocyte function, modulation of the *NDRG2* gene has been demonstrated in various preclinical studies to ameliorate astrocyte capability and even reduce the pathogenic effects of certain neurological disorders, demonstrating the potential therapeutic effects of astrocyte modulation [73].

#### 2.2.3 Abnormalities in Signaling Pathways and Energy Metabolism in PD

Abnormalities in astrocyte activation and related signaling pathways perform a vital function in PD. Zhang et al. [78] found that the *Cntnap4* gene served as a central factor in *α‐synucleinopathy* by regulating the C3–C3aR pathway between astrocytes and microglia. Furthermore, inhibition of MEK1/2 signaling reduces the inflammatory properties of astrocytes and helps to restore the integrity of the BBB, attenuates dopaminergic neuronal damage in PD by regulating the activation status of astrocytes and promoting the conversion from type A1 to type A2 [77]. Abnormal energy metabolism in astrocytes is equally important for the advancement of PD. It has been indicated that elevated NADPH oxidase (NOX4) acts in conjunction with inflammatory cytokines (e.g., MPO and OPN) in astrocytes in the hippocampus to induce abnormal mitochondrial function, which accelerates the development of PD [74]. Meanwhile, astrocytes in PD exhibit defective OXPHOS function, which may impair their supportive role for dopaminergic neurons, further leading to neuronal loss [75].

### 2.3 Huntington's Disease

HD is an autosomal dominant neurodegenerative disease [83], which is identified by motor dysfunction, cognitive disability, and psychiatric symptoms that increase over the course of the disease [83]. HD is a neurodegenerative ailment that stems from genetic mutations within the Huntington's protein (*Htt*) gene. This aberrant genetic alteration precipitates the buildup of mutant Huntington's protein (mHtt) in the cerebral cortex. The accrual of mHtt subsequently triggers neuronal deterioration, constituting the principal pathological hallmark of HD [84]. The pathogenesis of HD involves multiple molecular mechanisms, including mitochondrial dysfunction, oxidative stress, excitotoxicity, impaired proteostasis, and transcriptional dysregulation [85]. The pathophysiology of HD has yet to be fully investigated [86]. Research suggests that glial cell dysfunction occurs in the early stages of HD and that interventions targeting these cells may have therapeutic potential before motor and cognitive clinical manifestations emerge [87]. The subsequent chapter centers around the function of astrocytes in HD in relation to inflammatory responses, gene expression, abnormal electrophysiologic function, and metabolic abnormalities (Table 3).

**TABLE 3:** Molecules and mechanisms associated with astrocytes in Huntington's disease.

Key molecules	Implications	References
Potassium	Dysregulation of extracellular potassium ions ([K^+^]_e_) resulting in neurodegeneration	[88]
Ion change	Reduces support for neuronal development	[89]
PTL	Amelioration of motor, cognitive, and anxiety‐related symptoms, protection of neuronal integrity, promotion of astrocyte conversion to A2 phenotype, inhibition of NF‐κB and NLRP3 inflammatory vesicles	[90]
Glial cells	In HD patient, the fraction of activated microglia witnessed an increment, and this augmentation was in direct proportion to the burden of 1C2‐labeled mutant proteins.	[91]
JAK2–STAT3 pathway	Reduces mutant protein aggregation, improved neuronal function, and improves disease symptoms.	[92]
SREBP2 gene	Activates cholesterol synthesis genes, restores synaptic function, reverses the decline of dopamine receptor D2, cleares mutant protein aggregation, and improves behavioral defects	[93]
24S‐Hydroxycholesterol	An indicator for neurodegenerative disorders	[94]
Cholesterol synthesis gene	The expression of cholesterol synthesis genes is decreased in HD patients and models, especially in the striatum.	[95]
SCFAs	Improves the balance of intestinal flora, alleviates cognitive impairment, and regulates neural energy metabolism	[22]
Dopamine	Regulates the morphology and function of astrocytes	[96]
ROS	Exacerbates HD progression.	[97]
MSNs	Contributes to disease progression	[98]
GABA	Affects the inhibitory regulation of the CNS	[99]
Synaptic and extra synaptic glutamate and potassium ions	Triggers striatal hyperexcitability and promotes synaptic dysfunction	[100]
G(i)–GPCR pathway	Improves astrocyte, synaptic, and behavioral phenotypes associated with HD	[101]
ZFP inhibitor	Reduces mHTT expression in astrocytes	[102]
Cell cycle and p53 signaling pathway	The expression of these genes decreases in neural progenitor cells and increased in astrocytes.	[103]
ADGRL3	Mitigates HD progression	[104]
NeuroD1 and Dlx2 transcription factors	Transfigures astrocytes residing in the striatum of HD mice into GABAergic neurons	[105]

Abbreviations: ADGRL3, adhesion G protein‐coupled receptor L3; HD, Huntington's disease; MSNs, medium spiny neurons; PTL, apigenin; ROS, reactive oxygen species; SCFAs, short‐chain fatty acids; ZFP, zinc finger protein.

#### 2.3.1 Glial Cell Activity and Inflammatory Response in HD

Modulation of glial cell activity and inflammatory response is recognized as an effective approach to alleviate symptoms [90]. Studies have shown that apigenin (PTL) is able to alleviate inflammation by reducing the stimulation of NF‐κB and NLRP3 inflammatory vesicles and promotes the transformation of astrocytes to a protective A2 phenotype, thereby contributing to the improvement of neuronal health and enhancement of motor and cognitive function [90]. In addition, activation of microglia in HD patients showed a positive correlation with mutant protein load and emotional symptoms, further indicating an essential role of inflammation in the HD disease process [91]. Meanwhile, activation of the JAK2–STAT3 pathway significantly decreases the accumulation of mutant proteins, increases glutamate levels, and attenuates striatal atrophy by enhancing the protein degradation mechanism of astrocytes [92]. Under pathological conditions, astrocytes can release GABA, which may interfere with CNS inhibitory regulatory mechanisms [99]. In the meantime, the abnormal function of astrocytes in HD with respect to synapses leads to an overaccumulation of glutamate and potassium ions, which contributes to hyperexcitability in striatal regions, thus exacerbating the impairment of synaptic function [100]. It has also been shown that stimulation of the G(i)–GPCR pathway alters astrocyte responses, thereby improving HD‐related synaptic and behavioral phenotypes [101]. These findings suggest that integrally modulating the inflammatory response of glial cells and their capacity to eliminate mutant proteins might act as a substantial therapeutic method to alleviate the symptoms of HD.

#### 2.3.2 Gene Expression in HD

Gene expression plays an important role in HD. Diaz‐Castro et al. [102] found that reduction of mutant mHTT in astrocytes using a ZFP transcriptional inhibitor inverted expression changes of 61 core genes and partially restored normal function of astrocytes, which provided a new direction for therapies targeting HD. Meanwhile, Goodnight et al. [103] showed that during differentiation from neural progenitor cells (NPCs) to astrocytes, the expression patterns of genes related to the cell cycle and p53 signaling pathway were significantly altered in an HD model, suggesting that these genes occupy an important position in the functional development of astrocytes. Furthermore, Huynh et al. [104] noted that *OLIG2* and *TCF7L2* were identified as key regulators that exhibit a common pattern of transcriptional dysregulation in HD and schizophrenia, which might affect glial cell response to glutamatergic signaling, leading to insufficient myelin production and synaptic dysfunction. Finally, a gene therapy‐based approach for converting astrocytes into GABAergic neurons in HD mice shows the capacity to invert the advancement of HD [105].

#### 2.3.3 Abnormalities in the Electrophysiological Function of Astrocytes

Significant abnormalities in the electrophysiological function of astrocytes were observed in HD patients and animal models. Astrocytes from HD patients exhibited impaired potassium currents, prolonged calcium waves, and reduced membrane capacitance, which resulted in reduced neuronal support of astrocytes [89]. Potassium ions, as an important regulator of the electrophysiological stability of neurons, are dysfunctional in the potassium buffering function of astrocytes in HD, leading to an increase in the extracellular concentration of potassium ions, which directly affects the excitability of neurons, and consequently exacerbates neuronal damage [88]. Astrocytes maintain homeostasis in the extracellular environment through their ion channels, and when this function is disrupted, pathological changes result [88].

#### 2.3.4 Metabolic Abnormality in HD

Bennett Van Houten demonstrated that the metabolic pathway of astrocytes shifted from glycolysis to fatty acid oxidation in a mouse model of HD, and this metabolic abnormality increased oxidative stress, further exacerbated neuronal damage, and was closely associated with mitochondrial dysfunction [97]. It was also found that antioxidants such as XJB‐5‐151 could effectively inhibit the production of mitochondrial ROS and reverse the damage induced by oxidative stress on astrocytes and neurons, protecting the mice from damage [97]. In addition, cholesterol occupied a vital position in the development of HD. It was discovered that the expression of cholesterol‐synthesizing genes was reduced in HD patients and animal models, especially in the striatal region [106]. Reduced cholesterol synthesis is strongly associated with decreased synaptic function, which in turn affects neuronal function [94]. By delivering the *SREBP2* gene to astrocytes, cholesterol synthesis can be restored, synaptic function can be improved, mutant protein aggregates can be removed, and behavioral deficits can be ameliorated [93]. This study provides new ideas for treating HD by restoring cholesterol metabolism. In addition to cholesterol, fatty acids and dopamine also occupy a position in the development of HD. It has been shown that SCFA dietary supplementation alleviates cognitive deficits in AD mice by modulating intestinal flora and enhancing communication between neurons and astrocytes [22]. However, in the case of HD, dopamine can regulate IL‐6 expression and morphological changes in astrocytes via specific receptors, thereby regulating its functional effects [96].

### 2.4 Amyotrophic Lateral Sclerosis

ALS is a neurodegenerative disorder defined by the progressive degeneration of motor neurons in the brain and spinal cord [107]. While motor neurons are the primary cells affected in ALS, the contribution of astrocytes, a primary type of glial cell type in CNS, has been increasingly recognized as pivotal in both disease onset and progression [108].

Astrocytes provide metabolic, structural, and trophic support to neurons under physiological conditions [109]. In ALS, however, astrocytes undergo a reactive transformation that contributes to neuroinflammation, excitotoxicity, and oxidative stress, exacerbating motor neuron damage [110] (Table 4).

**TABLE 4:** Molecules and mechanisms associated with astrocytes in amyotrophic lateral sclerosis.

Key molecules	Implications	References
*Arf1*	IFN‐γ plays a key role in the neurodegenerative pathway triggered by *Arf1* ablation.	[111]
miR‐494‐3p	Protects motor neurons from degeneration	[112]
PGK1	PGK1 overexpression alleviated motor axon defects and enhanced motor function in ALS models.	[113]
TGF‐β1	TGF‐β1, upregulated in ALS astrocytes, inhibits neuroprotective inflammation.	[114]
*Egln2*	Downregulating *Egln2* protected motor neurons and reduced the ALS phenotype in animal models.	[115]
polyP	Excessive astrocyte‐derived polyP contributes to non‐cell‐autonomous motor neuron degeneration.	[116]
Cx43	Cx43 gap junctions and hemichannels facilitate astrocyte communication in the CNS and contribute to neurotoxicity in ALS.	[117]
IL‐1α, TNFα, and C1q	Knocking out IL‐1α, TNFα, and C1q significantly improves survival in an ALS mouse model.	[118]
EphB1	EphB1 triggers a protective, anti‐inflammatory response in astrocytes, partly through the STAT3 network.	[119]
EAAT2	Restoring EAAT2 levels in membralin KO astrocytes limited astrocyte‐dependent excitotoxicity in motor neurons.	[120]
IGF1R	Inhibiting the IGF1R–MTOR pathway reduces cell proliferation and reactivity in mutant *SOD1* astrocytes, decreasing their toxicity to motor neurons.	[121]
NEMO	Upon oxidative stress, NEMO is recruited to mitochondria in astrocytes. Damaged, ubiquitinated mitochondria trigger innate immune signaling.	[122]
TRIF	TRIF deficiency decreased the infiltration of NK cells, NK‐T lymphocytes, and CD8‐T cells in ALS mice.	[123]
RIPK1	Blocking RIPK1 kinase activity delays the onset of ALS and mitigates motor deficits, concurrently regulating astrocyte reactivity.	[124]

Abbreviations: Cx43, connexin 43; EAAT2, excitatory amino acid transporter 2; Egln2, Egl‐9 family hypoxia‐inducible factor 2; EphB1, ephrin type‐B receptor 1; IGF1R, insulin like growth factor 1 receptor; MTOR, mechanistic target of rapamycin kinase; NEMO, NF‐κB essential modulator; PGK1, phosphoglycerate kinase 1; polyP, polyphosphate; RIPK1, receptor‐interacting protein kinase 1; STAT3, signal transducer and activator of transcription‐3; TRIF, TIR domain‐containing adaptor inducing interferon‐β.

In familial ALS, astrocytes exhibit significant heterogeneity in their reactive states. *SOD1* mutant astrocytes primarily activate the NF‐κB pathway, leading to increased secretion of proinflammatory cytokines such as IL‐6 and IL‐8. In contrast, *VCP* mutant astrocytes show upregulation of IRF‐1 and MHC class I molecules, indicating potential antigen‐presenting capabilities. Notably, *VCP* mutant astrocytes autonomously express C3 without external stimulation, whereas C3 induction in *SOD1* mutants requires proinflammatory stimuli, suggesting a greater dependence on non‐cell‐autonomous mechanisms [125]. In the spinal cords of ALS patients, single‐nucleus RNA sequencing identified three distinct astrocyte subpopulations: Ast 1, Ast 2, and Ast 3. Homeostatic Ast 1 astrocytes were significantly reduced in ALS samples, while Ast 2 and Ast 3 populations were relatively enriched. The Ast 2 cluster showed upregulation of BHLHE40, a transcription factor regulating inflammation, and was associated with biological processes such as apoptosis. The Ast 3 cluster highly expressed reactive and inflammatory genes, including *CHI3L1*, *CHI3L2*, *CCL2*, *SERPINA3*, and *VIM*. These upregulated genes were enriched in processes such as wound response, chemotaxis, and cytokine signaling, and were strongly associated with ALS pathogenesis. Together, these findings suggest that astrocytes undergo disease‐associated state transitions and that their subpopulation heterogeneity may play a crucial role in driving neuroinflammation in ALS [124].

#### 2.4.1 Reactive Astrogliosis and Impaired Glutamate Clearance

Astrocytes in ALS undergo reactive astrogliosis, marked by cellular hypertrophy, increased proliferation, and elevated expression of GFAP [126]. This reactive state is influenced by inflammatory mediators such as TGF‐β, IL‐1β, and TNF‐α, which are elevated in ALS [118, 120]. RAs further compromise neuronal function by impairing synaptic glutamate clearance. Reduced expression of excitatory amino acid transporter 2 results in extracellular glutamate accumulation, overactivation of AMPA and NMDARs on motor neurons, and subsequent excitotoxicity—key mechanisms of motor neuron degeneration. These deficits have been linked to mutations in ALS‐associated genes, such as *SOD1* [120].

#### 2.4.2 Oxidative Stress and Mitochondrial Dysfunction

In ALS, astrocytes are major contributors to ROS. Mutant *SOD1* amplifies oxidative stress by disrupting mitochondrial function and elevating ROS levels, leading to further motor neuron damage [127]. Upon oxidative stress, NF‐κB essential modulator is recruited to mitochondria in primary astrocytes. Damaged and ubiquitinated mitochondria trigger mitophagy, disrupting function, and forming activated IKK complexes, which activate NF‐κB signaling and exacerbate cellular damage and inflammation [122].

#### 2.4.3 Chronic Inflammation and Toxic Feedback Loops

ALS astrocytes secrete proinflammatory cytokines and chemokines, promoting a chronic inflammatory state in the CNS. Activated microglia and infiltrating immune cells respond to astrocyte‐derived signals, creating a toxic feedback loop that accelerates motor neuron death. TGF‐β1 expression is elevated in astrocytes in both murine and human ALS models, and pharmacological inhibition of TGF‐β signaling post disease onset has been shown to prolong survival in *SOD1* mice [114]. Guttenplan et al. [118] further demonstrated that the combined knockout of IL‐1α, TNFα, and C1q in *SOD1*‐mutant mice effectively blocked the formation of inflammatory RAs and significantly extended the lifespan of ALS mice. Additionally, IFN‐γ, a key downstream factor in the neurodegenerative pathway induced by *Arf1* ablation, may activate the A1 astrocyte–C3 axis, contributing to neuronal and oligodendrocyte damage [111]. In mutant SOD1‐expressing astrocytes, a notable upregulation in the expression of the insulin‐like growth factor 1 receptor (IGF1R) is observed. This elevation in IGF1R levels triggers an augmented phosphorylation of AKT, which in turn leads to the subsequent activation of the mTOR signaling cascade. Sustained activation of the mTOR pathway suppresses autophagy, promotes cell proliferation, and enhances astrocyte reactivity. Suppressing the IGF1R–MTOR pathway has been found to mitigate their toxicity to motor neurons, highlighting its potential as a therapeutic target in SOD1‐associated ALS [121]. Furthermore, TRIF deficiency decreased infiltration of NK cells, NK‐T lymphocytes, and CD8‐T cells into the spinal cord of ALS mice, suggesting that the TRIF pathway plays a protective role in maintaining the motor neuron microenvironment by clearing aberrantly activated astrocytes [123].

#### 2.4.4 Exosome‐Mediated Propagation of ALS Pathology

Astrocytes release EVs containing mutant proteins or dysregulated microRNAs, playing a critical role in motor neuron injury and disease progression [128]. In vitro studies have shown that primary astrocytes overexpressing mutant SOD1 activate unconventional secretion pathways, resulting in increased release of EVs carrying mutant SOD1. These EVs can be internalized by motor neurons, leading to intracellular SOD1 aggregation and subsequent neuronal death [129]. Additionally, miR‐494‐3p is significantly downregulated in ADEVs, this microRNA normally suppresses the expression of SEMA3A, a protein whose upregulation is closely associated with neuronal degeneration and represents a common pathological feature in ALS. Notably, exogenous supplementation of miR‐494‐3p has been shown to reduce SEMA3A levels and rescue motor neurons from degeneration [112].

## 3 Astrocytes in Stroke

In 2019, the global stroke situation presented a grave picture. There were 12 million newly occurred strokes and 101 million prevalent ones, which cumulatively led to 143 million disability‐adjusted life years (DALYs). Globally, stroke ranked as the second major cause of mortality following ischemic heart disease and stood as the third principal contributor to both death and disability [130]. In China, there are more than 2 million new cases of stroke each year. It is the disease that causes the largest number of DALYs. Given the aging population, the persistently high prevalence of risk factors, as well as the insufficient treatment options available, the burden is projected to be exacerbated. Among other things, the risk factors for stroke are of great preventive value [131]. Risk factors for stroke can be categorized into noninterventional and interventional factors. The former include age, race, and genetics, while the latter cover hypertension, dyslipidemia, diabetes, heart disease, poor diet, smoking, alcohol consumption, obesity or overweight, physical inactivity, and psychological factors [132]. Management of interventional factors is a core strategy for stroke prevention, and controlling these factors can significantly decrease the likelihood of stroke and the burden of disease. In China, the incidence of stroke continues to rise, with significant regional and urban–rural differences. In urban regions, stroke care has witnessed notable advancements. Specifically, various interventions have been implemented with the key objective of diminishing the potential for the previously mentioned negative consequences. However, when it comes to rural areas, there remains a paucity of research data concerning treatment modalities and the ultimate outcomes of stroke patients. In addition, because data on stroke care in the community are not yet fully available, the organization of stroke care and the specific implementation of interventions are often unclear, and these issues warrant further study in the future [131, 133].

In conclusion, stroke has become a major challenge in global and Chinese healthcare, and further exploration of its pathogenesis and optimization of prevention and treatment are urgently needed. All sectors of society should pay more attention to the whole process of stroke prevention, treatment, and rehabilitation. Only through the concerted efforts of the whole world can this serious public health problem be effectively addressed.

### 3.1 Ischemic Stroke

**FIGURE 2:**
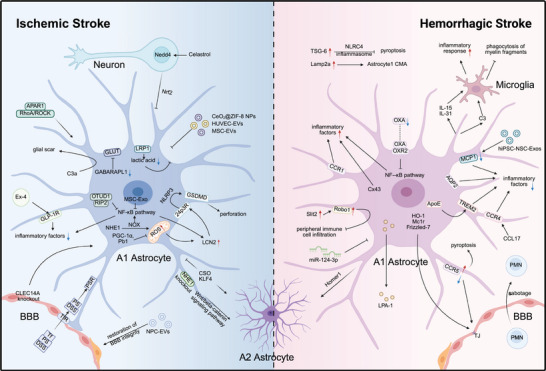
The mechanism of astrocytes in ischemic and hemorrhagic stroke. On the left are the molecular mechanisms associated with ischemic stroke and on the right are those associated with hemorrhagic stroke. The part of ischemic stroke mainly involves the mechanism of the oxidative stress, blood–brain barrier damage, metabolism, inflammation, the section of extracellular vesicles, and polarization of A1 and A2 type astrocytes. The part of hemorrhagic stroke mainly involves molecules related to inflammation, anti‐autophagy, damage to the integrity of the blood–brain barrier, and astroglia–microglial interaction. The figure was created using BioRender.com.

Ischemic stroke (IS) is a serious disease with complex pathophysiological mechanisms. IS occurs when the blood perfusion of brain tissues is interrupted or blocked, resulting in a decrease in the provision of oxygen and essential nutrients, and ultimately leads to necrosis of neuronal tissues [134]. Existing studies suggest that the mechanisms of IS include neuroinflammation, oxidative stress, BBB damage and repair, metabolic abnormalities, cell death, and so on. The majority of studies concentrate on the changes and roles of neurons; however, glial cells also play a crucial part in IS [135]. Following IS, astrocytes exhibit a dual nature, yielding both advantageous and disadvantageous consequences. In terms of the beneficial aspect, they confer neuroprotection and actively partake in neurorecovery. Poststrokely, they manifest robust neurorestorative capabilities by spurring angiogenesis, facilitating neurogenesis, fostering synaptogenesis, and driving axonal remodeling. Conversely, on the harmful side, they secrete inflammatory mediators that can intensify ischemic damage during the acute phase, posing potential challenges to the overall recovery process [136, 137]. In addition, in recent years, single‐cell RNA sequencing has provided new insights into the heterogeneity of subpopulations among various tissues. Bormann et al. [138] identified five astrocyte subsets by single‐nucleus RNA sequencing. Previously prescribed stable astrocytes continue to be divided into two subsets, AC_1 and AC_2. At the same time, RAs were also divided into three subsets, namely, AC_3, AC_4, and AC_5. Ma et al. [139] found that the heterogeneity of astrocytes in IS was not only limited to the traditional A1 versus A2 phenotype, but might also result from dynamic shifts in the characteristics of the same cell population. In the context of a mouse model of temporary middle cerebral artery occlusion, astrocytes tended to exert neuroprotective effects in localized ischemia–reperfusion areas at 12 hours, whereas astrocytes acted as signal amplifiers and released inflammatory signals that caused injury at 24 hours [139]. Consequently, delving into the accurate mechanisms by which astrocytes function in the context of IS is of great significance. It will empower us to gain a more thorough comprehension of the progression of the disease and open up the way for new therapeutic strategies in the future. The main mechanisms mentioned in the review can be seen in Figure 2.

#### 3.1.1 Oxidative Stress‐Related Roles Exerted by Astrocytes

IS is accompanied by significant oxidative stress, and astrocytes have a crucial part in this process. Studies have shown that adenosine deaminase (ADAR1), which is normally not expressed in astrocytes, is significantly induced at lesion sites after IS. ADAR1 induces astrocytes to secrete a sequence of inflammatory factors, such as TNF‐α, IL‐1β, and IL‐6, through the production of ROS, the release of which further triggers apoptosis of neurons, which ultimately aggravates the pathologic progression and prognosis of IS [140]. In addition, HIF‐1α is rapidly activated after ischemia and is widely involved in the pathophysiological process of IS as a key regulator of oxygen homeostasis. HIF‐1α has heterogeneous roles in different cell types, and in addition to regulating neuronal survival, neuroinflammation, angiogenesis, and BBB homeostasis, HIF‐1α holds a crucial position in the repair and inflammatory response to ischemic injury by regulating the metabolism and function of astrocytes [141]. In the context of oxidative stress regulation, peroxisome proliferator‐activated receptor‐γ coactivator‐1α (PGC‐1α) assumes a pivotal position. It has been ascertained that in a mouse model afflicted with vascular dementia resulting from chronic cerebral hypoperfusion, the overexpression of PGC‐1α can markedly enhance cognitive function and safeguard hippocampal neurons against ischemic harm. This is achieved through curtailing the production of ROS and suppressing the excessive activation of astrocytes and microglia [142]. In addition, Celastrol, a natural compound, is able to activate Nrf2 by binding to neuronally expressed developmentally downregulated 4, thereby inhibiting K48‐linked ubiquitination of Nrf2 degradation, reducing oxidative stress and inhibiting astrocyte hyperactivation, which ultimately protects neurons from axonal damage and apoptosis [143]. In addition, the RhoA/ROCK pathway has a significant part in axonal demyelination after ischemic injury. It was found that H_2_S promoted the conversion of astrocytes and microglia to beneficial subtypes by inhibiting the upregulation of the RhoA/ROCK pathway, which further promoted the regeneration of myelin sheaths, and consequently improved the neurological recovery in IS [144].

In general, oxidative stress holds a crucial position in the pathological progression of IS. It exerts its influence mainly by regulating the activated state of astrocytes and the subsequent inflammatory reactions they trigger. Through the regulation of pivotal elements like ADAR1, HIF‐1α, PGC‐1α, Nrf2, as well as RhoA/ROCK, novel concepts and therapeutic approaches for the management of IS might be uncovered, potentially opening up new avenues for effective treatment.

#### 3.1.2 Coordinated Role of Astrocytes on BBB Permeability

After IS, the BBB faces severe disruption, and the role of neuroglia is particularly critical. During the acute phase, peripheral immune cells such as monocytes, neutrophils, and T‐lymphocytes significantly increase the permeability of the BBB by causing microvascular disturbances and secreting inflammatory factors. The active involvement of these cells exacerbates the early BBB damage, but over time, the role of these immune cells gradually shifts and they begin to participate in the restoration procedure of the BBB, facilitating angiogenesis and restoration of barrier function. Astrocytes and microglia, among others, also play an important immunological role in this process, and these cells not only respond to inflammatory signals but also assist in the repair of damaged BBB structures through a variety of mechanisms [145]. In recent times, the significance of astrocytes in the restoration of BBB has been drawing ever more focus. According to the experimental results of Bormann's team [138], AC_3 and AC_4 are involved in the reconstruction of the BBB, the remodeling of the extracellular matrix, and the guidance of neuronal and glial cell migration. This plays an important role in wound healing and neuroprotection. AC_5 may be involved in cell signaling and cell motility to repair severe brain damage [138]. Song and colleagues [146] provided evidence indicating that astrocytes devoid of the pH‐sensitive Na^+^/H^+^ exchange protein Na^+^/H^+^ exchanger 1 (NHE1) had the capacity to trigger the Wnt/β‐catenin signaling pathway following acute ischemia. This activation, in turn, promoted the process of BBB repair. After transforming into a “protective” phenotype, these astrocytes enhanced angiogenesis and cerebral perfusion by upregulating Wnt7a expression, providing important support for the restoration of the damaged BBB [146]. In addition, lipid carrier protein‐2 (LCN2) also plays a crucial role in the process of BBB damage and restoration. Following acute ischemic injury, LCN2 expression was induced in brain endothelial cells, neutrophils, and astrocytes, and further promoted BBB injury and associated inflammatory responses. By specifically targeting monoclonal antibody to LCN2, it can effectively attenuate brain injury, inhibit BBB leakage, and slow down cerebral edema and immune cell infiltration, and these results indicate that LCN2 has the potential to be an effective target for the therapy of BBB injury [147].

Overall, astrocytes, immune cells, and various other cell types assume intricate and essential functions in both the damage and restoration of the BBB following IS. A profound comprehension of these underlying mechanisms could furnish a significant theoretical foundation for devising novel therapeutic approaches, particularly with respect to enhancing the recovery of BBB functionality.

#### 3.1.3 Metabolism of Astrocytes

In IS, metabolic dysregulation plays a key role in neurological injury and recovery. It was found that SWELL1, a subunit of volume‐regulated anion channels (VRACs), has a dual role in ischemic injury. Activation of SWELL1 channels in neurons causes cell swelling and glutamate excitotoxicity, which aggravates neuronal injury. At the same time, SWELL1 in astrocytes is also involved in pathologic glutamate release, which increases the frequency of NMDARs currents and leads to worsening of nerve injury. Inhibitors targeting SWELL1 attenuate these cytotoxic responses, provide neuroprotection, and significantly alleviate cell death [148]. In addition, SHPL‐49 enhances glutamate recycling through augmenting the expression of glutamate transporter protein‐1 and activates the NMDA receptor subunit NR2A, resulting in neuroprotection in ischemic injury. The mechanism of action of SHPL‐49 includes protection of CaMKIIα from hypoxia–ischemia‐induced autophosphorylation injury, which contributes to a prolonged therapeutic time window and improves postischemic functional recovery [149].

The glycolytic function of astrocytes is also severely affected after IS. Downregulation of GABA type A receptor‐associated protein 1 (GABARAPL1) in IS patients and mouse models led to dysfunctional glycophagy in astrocytes. This dysfunction of glycophagy was further exacerbated through the activation of the PI3K–Akt pathway, which affected the O‐GlcNAcylation levels of transcription factor‐specific protein 1 and TATA‐binding proteins and inhibited their nuclear translocation. This process led to oxidative damage and DNA damage in astrocytes, and restoration of GABARAPL1 expression significantly improved glycophagy, reduced cellular damage and increased neuronal survival [150]. In addition, miR‐210, as an important microRNA molecule, its expression in astrocytes was regulated by ischemic and stress conditions. It was shown that miR‐210 enhanced the tolerance of astrocytes to ischemia by promoting glycolysis. Upregulation of miR‐210 upon activation of HIF‐1 improved ischemic tolerance in brain tissues and yields a fresh potential target for the curing of acute IS [151]. Lactate, a key molecule in astrocyte metabolites, also has an impact on IS. Lactate produced by astrocytes exacerbates brain damage by facilitating the formation of the protein lysine lactate (Kla). Suppression of lactate production or Kla formation in the lactate metabolic pathway significantly attenuates neuronal death and glial cell activation, thereby prolonging the reperfusion window and enhancing functional rehabilitation. This suggests that lactate production and its metabolic pathway may hold a prominent place in IS [152].

Imbalanced lipid metabolism also has a significant impact on the pathogenesis of IS, and long‐chain acylcarnitines (LCACs) hold an important position in IS. Accumulation of LCACs further damages neurons by inducing mitochondrial dysfunction in astrocytes. Imbalanced metabolism of LCACs not only affects neuronal energy supply, but also exacerbates cerebral perfusion deficiency and brain tissue damage. By interfering with the accumulation of LCACs, it may help to improve the neuroprotective effect in IS [153]. LRP1 is an endocytosis/signal transduction cell surface receptor that regulates a variety of cellular functions, including cell survival, differentiation, and proliferation. In an IS mouse model, inhibition of astrocyte LRP1 reduced mitochondrial translocation to damaged neurons and exacerbated ischemia–reperfusion injury [154]. In addition, the ketogenic diet mimetic β‐hydroxybutyrate (βHB) demonstrated positive effects in metabolic recovery after stroke. Long‐term administration of βHB activates the Nrf2/ARE pathway, enhances the expression of mitochondrial antioxidant genes, and accelerates the recovery of brain tissues by promoting neuronal plasticity, providing additional energy support, and reducing the neuroinflammatory response. βHB not only occupies a significant position in neuroprotection after mitochondrial injury, but also supports the functional restoration of neurons by improving the metabolic state [155]. In addition, by performing single‐cell RNA sequencing of astrocytes in the subacute stroke phase in mice, Scott et al. [156] found that astrocytes at the near‐injury site exhibited differential expression of genes associated with lipid shuttling, such as enrichment of *Apoe* and *Fabp5*.

The mitochondrial function of astrocytes is also closely related to neuronal survival. Ni et al. [157] found that the ginsenoside Rb1 protected mitochondrial function by decreasing ROS production, inhibiting NADH dehydrogenase in the mitochondrial complex I, and preventing reverse electron transfer and ROS generation, which further inactivated astrocytes. Furthermore, Rb1 promoted the translocation of mitochondria from astrocytes toward neurons. As a consequence, it augmented the membrane potential as well as the oxygen consumption rate of neurons, and consequently contributed to the improved survival of neurons [157].

In summary, metabolic alterations occupy an important place in all aspects of IS. Dysregulation of pathways such as VRACs, glucose metabolism, lipid metabolism, and lactate production not only exacerbate neurological damage, but also provide new targets for intervention in neuroprotection. These research results offer a substantial theoretical foundation for the management of IS and disclose multiple prospective metabolic intervention approaches.

#### 3.1.4 Relationship Between Astrocytes and Inflammation

Inflammation runs through the complete cycle of the origination and expansion of IS and forms part of the main mechanisms of cerebral injury [158]. The inflammatory response in the brain activates astrocytes. And A1 astrocytes, in turn, exacerbate the inflammatory response in the brain, this creates a vicious circle. However, A2 astrocytes tend to exert anti‐inflammatory and neuroprotective effects.

So how do astrocytes polarize after a stroke? Wang and Li [159] demonstrated that KLF4 has a significant impact on regulating the direction of astrocyte activation after IS. The increase of KLF4 content could inhibit the phosphorylation of NF‐κB on the one hand, thereby inhibiting the activation of A1 astrocytes, and on the other hand, it could facilitate the polarization of A2 astrocytes [159]. Liu and colleagues [160] found that cottonseed oil (CSO) could modulate the polarization direction of type A1 and type A2 astrocytes after stroke. CSO repressed the production of A1 astrocytes and promoted the polarization of A2 astrocytes, which could inhibit the expression of TLR4 and NF‐κB proteins, thereby reducing the release of IL‐1β, IL‐6, and TNF‐α [160]. In addition, astrocytes that are activated as type A1 can also be further converted to type A2 to minimize the inflammatory response. Bai et al. [158] used the principle that transferrin (TF) targets the overexpressed TF receptor on BBB to carry DSS through the TF and phosphatidylserine (PS)‐modified liposomes (TF/PS/DSS‐LPs) across the BBB. TF/PS/DSS‐LPs are taken in by activated astrocytes mediated by PS receptors, thereby converting astrocytes from A1 to A2 status to reduce neuroinflammation and neuronal damage [158]. Xian et al. [161] found that mesenchymal stem cell‐derived exosomes (MSC‐Exo) could reduce the transition of astrocytes to type A1. MSC‐Exo was able to significantly reduce the increased expression of TNF‐α and IL‐1β attributed to LPS‐stimulated astrocytes through the NF‐κB‐Nrf2 signaling pathway [161].

Astrocytes can secrete inflammatory factors and aggravate disorders in the brain. Zheng and colleagues [162] found that OTUD1 levels in astrocytes increased after the occurrence of IS. It interacted with receptor‐interacting kinase 2 (RIP2) and removed the polyubiquitin chain at the K63 position on RIP2, which inhibited the NF‐κB pathway, thereby reducing the secretion of IL‐6 and TNF‐α. Liu et al. [163] found that the amount of NHE1 in RAs increased after stroke, which activated NOX. On the one hand, it could promote the NF‐κB pathway, and on the other hand, it could increase the level of ROS. Both of these could augment the content of LCN2, which could damage neurons and lead to neurodegeneration. They also found that inhibitors such as HOE642 (NHE1 inhibitor) or DPI (NOX inhibitor) significantly reduced ischemia‐mediated NOX activation and ROS formation in vitro, which could be a new neuroprotective strategy [163]. Inflammatory factors secreted by astrocytes can act on themselves and exacerbate the inflammatory response. Li et al. [164] found that after ischemia/reperfusion injury in stroke, astrocytes secreted LCN2. LCN2 binds to the LCN2 receptor 24p3R on the cell membrane of astrocytes and activates the NLRP3 inflammasome. This allows the presence of gasdermin D (GSDMD) on astrocyte cell membrane to be demonstrated and causes membrane perforation, leading to astrocyte apoptosis. At the same time, IL‐1β and IL‐18 are released from the membrane pores composed of GSDMD, exacerbating the inflammatory response. Whereas, astrocyte‐specific AAV (AAV–GFAP–24p3Ri) can inhibit 24P3R, thereby reducing astrocyte pyroptosis. This can reduce the inflammatory response [164]. Bormann et al. [138], based on the results of single‐nucleus RNA sequencing, found that CD44‐positive RAs were significantly increased around the infarct lesion. These cells may interact with immune cells to repair brain damage through CD44 receptors involved in cell migration and inflammatory responses [138].

Activated astrocytes may alter the permeability of the BBB through an inflammatory response. Shan et al. [165] found that exendin‐4 can act on GLP1R on astrocytes in the case of oxygen–glucose deprivation, thereby reducing the excretion of inflammatory cytokines VEGF‐A and MMP‐9 and preserving the integrity of BBB. Endothelial cells constitute an integral part of the BBB and exhibit a connection with astrocytes as well. Kim and colleagues [166] made a discovery that when C‐type lectin family 14 member A (CLEC14A) was knocked out in endothelial cells, there was a remarkable augmentation in the quantity of GFAP^+^ cells within the ischemic zone of stroke. Concurrently, astrocytes underwent extensive activation, which in turn intensified the elevation of BBB permeability. This permitted a greater influx of inflammatory factors and cells into the cerebral milieu, ultimately culminating in nerve injury [166].

How to inhibit the overactivation of astrocytes is a vital approach to subduing the inflammatory reaction in the brain. Besides the interactions between biomolecules that have been discussed, new materials and technologies are also very important. He et al. [167] demonstrated that zeolitic imidazolate framework‐8–capped ceria nanoparticles (CeO_2_@ZIF‐8 NPs) possessed the capability to effectively prevent the activation of astrocytes that occurred during ischemia–reperfusion injury, thereby reducing further damage caused by inflammation. While inhibiting astrocyte activation and alleviating inflammatory responses, CeO_2_@ZIF‐8 NPs also demonstrated a high safety profile, offering a novel approach to the neuroprotective therapy of IS [167].

#### 3.1.5 Effect of ADEVs

EVs have shown a number of significant advantages in the treatment of IS—the biggest advantage is their low immunogenicity, which means that there is less risk of triggering an immune response during application. In addition, EVs have a low risk of vascular blockage and microvascular thrombosis, ensuring their safe circulation in the body. After systemic transplantation, EVs have exhibited remarkable prowess in crossing the BBB, allowing the molecules carried by EVs to act directly on brain lesions. At the same time, EVs are easy to mass‐produce and meet the urgent needs of clinical treatment. EVs have a higher surface area‐to‐volume ratio, which enhances the transfer efficiency of active particles to the target tissue. More remarkably, the miRNAs contained in EVs can be genetically modified with relative ease, which opens up more possibilities for personalized treatment [168]. Otero‐Ortega and their research group [169] ascertained that, subsequent to the astrocytes undergoing treatment via EV, the GFAP concentrations therein were conspicuously declined. Parallel to this, neurons in the medium‐dose cohort exhibited a substantially augmented level of synaptophysin [169]. It is reasonable to suspect that EVs can act on astrocytes to alter their morphology and function, ultimately promoting neuronal recovery. Is it possible to reduce the neuroinflammatory response through inhibiting the activation of astrocytes, so as to achieve neuronal recovery? Gao and colleagues [170] discovered that human umbilical vein endothelial cells‐derived extracellular vesicles (HUVEC‐EVs) were able to target astrocytes. HUVEC‐EVs carrying miR‐155‐5p can modulate the c‐Fos/AP‐1 signaling pathway in astrocytes, thereby improving the functional status of astrocytes, helping to reduce neuroinflammation, promote nerve regeneration, and repair after stroke [170]. Dabrowska et al. [171] found that intra‐arterial injection of MSCs‐EVs had a comparable effect to MSCs transplantation. Arterial injection of EVs significantly reduced the number of GFAP^+^ cells and inhibited astrocyte activation, thereby reducing neuroinflammation [171, 172]. The function of NPC‐derived extracellular vesicles (NPC‐EVs) in repairing the BBB has also been somewhat recognized [172]. Zhang et al. [173] found that under the conditions of OGD and LPS, NPC‐EVs were able to enter the BBB, interact with the terminal foot of astrocytes, reduce the activity of ATP‐binding cassette subfamily B member 1 transporter (ABCB1) and the activation of NF‐κB pathway, thereby alleviating the damage of the BBB [173].

### 3.2 Hemorrhagic Stroke

Hemorrhagic stroke represents a prevalent and severe subtype of stroke characterized by high rates of mortality and disability, divided into intracerebral hemorrhage (ICH) and subarachnoid hemorrhage [174]. The condition induces mechanical damage and hematoma formation, resulting in a significant increase in intracranial pressure, which is considered a primary injury. Subsequently, the degradation of blood components triggers secondary brain injury, manifesting as prolonged neuroinflammation, neuronal apoptosis, and disruption of BBB [175, 176]. Emerging evidence highlights the critical role of astrocytes in the pathophysiology of hemorrhagic stroke, exhibiting diverse mechanisms that influence inflammation, neuroprotection, and tissue repair. Their phenotypic heterogeneity is increasingly recognized, reflecting their varied roles and actions. Studies have shown that scRNA‐seq analysis of astrocytes in hemorrhagic mice identified ten functionally distinct subtypes. Subtype 3, characterized by the expression of *Txnip*, *Rbp1*, and *Sox9*, was enriched in ischemic brains and exhibited strong synapse‐clearing capacity, but was nearly absent in hemorrhagic brains. This loss may underlie the reduced phagocytic ability of astrocytes after hemorrhagic stroke. Additionally, astrocytes in hemorrhagic brains exhibited a downregulation of phagocytosis‐related genes such as *Hspa1a*, *Vim*, and *Mt1*, along with reduced expression of phagocytic vesicles and synaptic structures, indicating impaired synaptic clearance. This astrocyte heterogeneity may hinder synaptic repair and neural remodeling after hemorrhagic stroke, highlighting the importance of targeting astrocyte functional states for therapeutic intervention [177].

#### 3.2.1 Neuroinflammation and Immune Regulation

Astrocytes mediate inflammation and immune responses through the production of chemokines and cytokines. After ICH in mice, the endogenous expression of CCR1, CCR5, and CCL5 was significantly upregulated. Activation of CCR1 promoted neuroinflammation through CCR1/TPR1/ERK1/2 signaling pathway [178]. In addition, CCR5 activation has been shown to exacerbate neuronal pyroptosis and neurological impairments in ICH models, partly via the CCR5/PKA/CREB/NLRP1 signaling cascade [179]. Yan et al. [180] reported that blocking CCR5 ameliorated neurological deficits following ICH by maintaining BBB integrity through suppression of the CCR1/SRC/Rac1 pathway. Additionally, activation of the CCL5/CCR5 axis in various inflammatory cells, such as microglia, astrocytes, and monocytes, intensifies BBB disruption and neurobehavioral deficits post‐ICH, likely involving the JAK2/STAT3 signaling pathway [181]. Connexin 43 (Cx43), predominantly expressed in astrocytes, regulates astrocytic network homeostasis. Abnormal Cx43 expression was found to exacerbate inflammation, and its suppression exhibited anti‐inflammatory and neuroprotective effects post‐ICH [182]. Deng et al. [183] reported that the serum aquaporin (AQP) 2 levels were significantly reduced in ICH patients. Overexpression of AQP2 in GFAP‐labeled astrocytes in rats promoted astrocyte activation and secretion of proinflammatory cytokines, indirectly inducing microglial polarization. Serum AQP2 levels were inversely correlated with early‐stage ICH prognosis, suggesting its potential as an early marker of inflammation [183]. Moreover, transient receptor potential vanilloid 1 (TRPV1), a nonselective cation channel with high calcium permeability, has been associated with both neuronal apoptosis and the regulation of inflammatory processes. TRPV1 expression increased in neurons, astrocytes, and microglia following ICH. Genetic deletion of TRPV1 significantly mitigated motor deficits, neuronal apoptosis, brain edema, BBB permeability, and cytokine production within 1 day following ICH, highlighting its potential as a therapeutic target [184].

#### 3.2.2 Neuroprotection and Anti‐apoptotic Mechanisms

Astrocytes support neuronal survival and mitigate apoptosis by modulating specific signaling pathways. After ICH, the expression of Orexin A (OXA) was reduced. OXA supplementation improved neurofunctional outcomes, alleviated brain edema, and shifted the inflammatory balance toward anti‐inflammatory cytokines via the OXR2/CaMKKβ/AMPK pathway [185]. Moreover, Slit2, an extracellular matrix protein, exerts a repellent effect on leukocyte chemotaxis by interacting with the roundabout1 (Robo1) receptor. Li et al. [186] identified Robo1 expression on neurons, astrocytes, and infiltrating peripheral immune cells within the brain. Recombinant Slit2 reduced neuroinflammation by suppressing brain peripheral immune cells infiltration and neuron apoptosis after germinal matrix hemorrhage [186]. Similarly, activation of CCR4 and TREM2, which colocalized with astrocytes, neurons, and microglia, was associated with reduced brain edema, neuroinflammation, and neuronal apoptosis [187, 188]. Prior research has demonstrated that tumor necrosis factor‐stimulated gene‐6 (TSG‐6) contributes to neuroprotection by mitigating oxidative stress and inhibiting apoptosis. Ding et al. [189] discovered that TSG‐6, predominantly expressed in astrocytes, alleviated brain edema and neurological deficits via downregulating the expression of NLRC4 and pyroptosis of astrocytes.

MiR‐124‐3p was shown to inhibit neurotoxic activation of RAs by reducing saturated lipid secretion [190]. Autophagy also played a protective role following ICH, with Lamp2a, a critical regulator of chaperone‐mediated autophagy, being highly expressed in astrocytes. Downregulation of Lamp2a resulted in increased conversion of astrocytes into the neurotoxic A1 phenotype, exacerbating neuroinflammation [191]. Similarly, the scaffolding protein Homer1, localized in astrocytes, peaked 3 days post‐ICH and was found to suppress the transition of astrocytes to the A1 phenotype. Homer1 facilitated the induction of A2 astrocytes, which promoted neuroprotection and repair [192]. Fei et al. [193] further developed a method to generate Homer1a^+^ EVs derived from A2 astrocytes, enhancing stability, safety, and specificity for targeting injured neurons. These findings provide new insights and translational potential for Homer1a^+^ EVs in treating ICH.

#### 3.2.3 BBB Integrity

The breakdown of BBB represents a primary mechanism contributing to secondary brain damage following ICH. In a hemorrhagic stroke mouse model, a population of inflammatory RAs (GFAP^+^/Vim^+^/Lcn2^+^/Serpina3n^+^/C3d^+^) was identified, whose gene expression partially overlapped with neurotoxic A1 and disease‐associated astrocytes, suggesting functional crosstalk. These inflammatory RAs exhibit high expression of matrix metalloproteinase‐3 (MMP3), which directly disrupts BBB by degrading tight junction proteins and promotes the maturation of MMP9, thereby exacerbating vascular basement membrane damage [194]. Frizzled‐7, an essential protein expressed on endothelial cells, is crucial for controlling vascular permeability. Astrocytes expressed Frizzled‐7 after ICH, which significantly reduced BBB permeability to macromolecules [195]. Additionally, the expression of melanocortin‐1 receptor (Mc1r) increased after ICH, mainly expressed in microglia, astrocytes, and endothelial cells. Knockdown of Mc1r nullified its protective effects on BBB integrity at 24 hours post‐ICH [196]. Studies have shown that selective heme oxygenase‐1 (HO‐1) overexpression in astrocytes decreases mortality and mitigates BBB disruption in ICH models [197, 198]. Conversely, Wang et al. [199] discovered that preserving the integrity of BBB might be achieved by suppressing the production of monocyte chemoattractant protein‐1 through the activation of the PI3K/AKT signaling pathway in astrocytes. Furthermore, astrocytes exhibited expression of lysophosphatidic acid receptor post‐ICH, which exacerbated BBB disruption by promoting polymorphonuclear leukocyte recruitment via the TSP1/CXCR2 signaling pathway in mice [200].

#### 3.2.4 Astrocyte–Microglia Crosstalk

Astrocytes and microglia exhibit a dynamic interplay that coordinates their functions during hemorrhagic stroke. The expression of complement component C3 is significantly elevated in A1 RAs. At the same time, microglia display abundant levels of C3aR. This suggests that A1 astrocytes could play a role in facilitating microglial phagocytosis of myelin debris through the interaction between astrocytic C3 and microglial C3aR [201]. Moreover, IL‐15 expression is significantly upregulated in astrocytes from ICH patients, predominantly influencing microglial responses rather than those of other immune cell subsets. IL‐15 skews microglial activity toward a proinflammatory phenotype, thereby exacerbating neuroinflammation following ICH [202]. Jiang et al. [203] discovered IL‐31 levels in lesion sites of elderly ICH patients were significantly higher than in younger patients. Activated astrocytes post‐ICH release substantial amounts of IL‐31, which binds to microglia through the IL‐31 receptor (IL‐31R), resulting in the recruitment of microglia to the hematoma site. This recruitment amplifies neuroinflammation, induces neuronal apoptosis, and ultimately worsens functional recovery outcomes [203].

## 4 Astrocytes in MS

In MS, a persistent autoimmune disease characterized by neuroinflammation, demyelination, and neurodegeneration, astrocytes undergo significant functional changes, which often exacerbate disease pathology [204]. Studies using advanced tools like RABID‐seq have revealed diverse astrocyte subtypes with distinct transcriptional and functional profiles, interacting dynamically with microglia and other CNS cells during MS progression [205]. In addition, Itoh et al. [206] employed RiboTag technology to perform cell type‐specific and region‐specific transcriptomic analyses of astrocytes across various regions of the CNS. They found that astrocytes exhibited significant transcriptomic heterogeneity under disease conditions in regions such as the spinal cord, cerebellum, cortex, and hippocampus. Notably, in white matter regions, genes involved in cholesterol synthesis were markedly downregulated, while immune‐related genes were upregulated [206]. Trobisch et al. [207] further elucidated the heterogeneity of astrocytes across gray and white matter regions, identifying five major astrocyte subtypes. Among them, AS‐*GPC5* and AS‐*DPP10* were predominantly derived from gray matter, while AS‐*CD44* was mainly found in subcortical and spinal cord white matter. In the context of MS, AS‐*CD44* astrocytes in white matter exhibited distinct reactive features. Differential gene expression analysis revealed downregulation of genes involved in neuronal development and synaptic function, such as *ARL17B*, *HES1*, and *SRPX2*, specifically in spinal cord astrocytes. In contrast, stress‐related genes including *HSP90AA1*, *HSPB1*, and *FTL* were upregulated, indicating a highly reactive and stress‐responsive state. Notably, this subcortical white matter astrocyte subtype also showed high expression of *CD44* and *CPAMD8*, which might hinder remyelination by inhibiting the differentiation of oligodendrocyte precursor cells [207]. These findings suggest that astrocytes play crucial roles in inflammatory responses and their region‐specific metabolic alterations may impact myelin repair and neuroprotection, providing potential new targets for precision therapies in MS.

Activated astrocytes are central players in the inflammatory and neurodegenerative processes of MS. Upon activation, astrocytes release proinflammatory cytokines and chemokines, which exacerbate immune cell infiltration into the CNS, amplifying inflammation and neuronal damage [208]. The traditional view held that astrocytes primarily contributed to glial scar formation during the later stages of MS lesions. However, emerging evidence suggests that astrocytes become highly active at early stages of lesion development, where they phagocytose myelin debris and facilitate the release of chemokines. This response may aid in damage clearance, but it also has the potential to excessively activate immune responses and exacerbate disease progression [209]. Here are specific molecular mechanisms involving astrocytes in MS, aimed at identifying therapeutic targets for the disease (Table 5).

**TABLE 5:** Molecules and mechanisms associated with astrocytes in multiple sclerosis.

Key molecules	Implications	References
CD38	Regulates the activation of proinflammatory transcriptional networks	[210]
Dectin‐1	Facilitates advantageous interactions between myeloid cells and astrocytes via Osm‐OsmR signaling, mitigating CNS inflammation	[211]
CLU	Impairs astrocyte‐mediated clearance of myelin debris through inhibiting PI3K–AKT signaling in primary OPCs, thereby hindering remyelination by OPCs	[212]
CHI3L1	Astrocyte‐specific CHI3L1 deletion restores neurogenesis and ameliorates cognitive deficits.	[213]
FTY720	Mitigates neuroinflammation through regulation of B_12_ pathways in astrocytes	[214]
TrkB	Damages oligodendrocytes and causes myelin loss	[215]
Cav1.2 channels	Inhibition of Cav1.2 channels in astrocytes during EAE reduces neuroinflammation and safeguards the spinal cord against autoimmune demyelination.	[216]
YAP	Protects the optic nerve and retina from neuroinflammation and demyelination in EAE mice by enhancing the TGF‐β signaling pathway	[217]
IL‐3	Recruits immune cells into the CNS, thereby aggravating MS and neuroinflammation	[208]
XBP1	The interplay between XBP1 and NR3C2–NCOR2 pathways regulates the pathogenic activities of astrocytes, influencing CNS damage in EAE and potentially in MS.	[218]
MAFG	Promotes MAFG expression along with proinflammatory transcriptional pathways	[219]
TUDCA	Inhibits the polarization of astrocytes into neurotoxic phenotypes likely through its action on GPBAR1	[220]
HB‐EGF	Regulates tissue protection and inflammation in autoimmune neuroinflammation	[221]
LIF and TGF‐β1	The upregulation of neurotrophic factors in BMS astrocytes cocultured with neurons is induced by TNF‐α/IL‐17A through the JAK pathway.	[222]
Nrf2	Activation of astrocytic Nrf2 is linked to oligodendrocyte cell death and failure of remyelination.	[223]
RIPK1	Triggers an inflammatory transcriptional response that negatively affects oligodendrocytes	[224]
C3	C3 expression is increased in astrocytes of EAE mice. Conditional deletion of C3 in astrocytes protects RGCs.	[225]
LAMP1 and TRAIL	Mitigates inflammation in EAE by promoting T cell apoptosis	[226]
IRE1α and XBP1	Regulates the proinflammatory response of microglia and monocytes	[227]
IL‐27	Modifies the immune functions of human astrocytes and influences the profile and movement of T lymphocytes	[228]
PD‐L1	Induces leukocyte apoptosis and diminishes inflammation via upregulation of PD‐L1	[229]

Abbreviations: B12, vitamin B12, FTY720, fingolimod; BMS, benign multiple sclerosis; CHI3L1, chitinase‐3‐like; CLU, clusterin; EAE, experimental autoimmune encephalomyelitis; GPBAR1, G protein‐coupled bile acid receptor 1; HB‐EGF, heparin‐binding EGF‐like growth factor; IRE1α, inositol‐requiring enzyme‐1α; LAMP1, lysosomal‐associated membrane protein 1; NCOR2, nuclear receptor corepressor 2; NR3C2, nuclear receptor subfamily 3 group C member 2; OPCs, oligodendrocyte precursor cells; Osm, oncostatin M; OsmR, Osm receptor; PD‐L1, programmed death ligand 1; RGCs, retinal ganglion cells; RIPK1, receptor interacting protein kinase 1; TRAIL, TNF‐related apoptosis‐inducing ligand; XBP1, X‐box binding protein 1; YAP, yes‐associated protein.

### 4.1 Astrocytic Proinflammatory Mechanisms

Experimental autoimmune encephalomyelitis (EAE) is an inducible inflammatory disorder of CNS commonly employed as a preclinical model to study the pathogenesis and therapeutic strategies of MS in humans [230]. Astrocytes contribute to CNS inflammation by expressing molecules and activating signaling pathways that drive immune cell recruitment and cytokine production. Among these, CD38 upregulation in RAs is critical for neuroinflammation, while its inhibition enhances NAD^+^ levels, mitigates inflammatory responses, and improves outcomes in EAE models [210]. Moreover, scRNA‐seq analyses have revealed distinct astrocyte subtypes characterized by reduced NRF2 expression and elevated MAFG expression, resulting in increased DNA methylation and the downregulation of antioxidant and anti‐inflammatory pathways. These changes, driven by granulocyte‐macrophage colony‐stimulating factor signaling, result in CNS inflammation and pathology [219]. It has been discovered that CNS‐resident astrocytes generated IL‐3. IL‐3 signaling induces chemotactic programs in IL‐3Rα^+^ myeloid cells, enhancing immune cell recruitment [231]. Similarly, knocking down X‐box binding protein 1 (XBP1) in astrocytes has been shown to decrease the infiltration of peripheral inflammatory monocytes into the CNS [227]. In MS, astrocytic receptor interacting protein kinase 1 (RIPK1) is upregulated and drives neuroinflammatory responses, creating a neurodegenerative environment. Inhibiting RIPK1 kinase could attenuate EAE severity and disease progression [224].

### 4.2 Astrocytic Anti‐Inflammatory Functions

While astrocytes exacerbate neuroinflammation through specific pathways, they also exhibit protective functions under certain conditions. Deerhake et al. [211] demonstrated that Dectin‐1 activated Oncostatin M (Osm) and other neuroprotective molecules in EAE by driving myeloid cell‐astrocyte crosstalk via a Card9‐independent pathway, highlighting a potential therapeutic target for autoimmune neuroinflammatory diseases. Notably, a specific population of astrocytes expressing lysosomal‐associated membrane protein 1 and TNF‐related apoptosis‐inducing ligand (TRAIL) mitigates inflammation in EAE by promoting T cell apoptosis. This regulatory astrocyte population is maintained under homeostatic conditions by IFN‐γ from meningeal NK cells. However, during neuroinflammation, the expression of TRAIL in astrocytes is depressed by T cells and microglia [226]. Similarly, the upregulation of programmed death ligand 1 reduces inflammation by inducing leukocyte apoptosis via IFN‐γ signaling [229]. Additionally, astrocytic yes‐associated protein is proposed to alleviate neuroinflammation and demyelination in the optic nerve and retina of EAE mice via TGF‐β signaling pathways [217]. Interestingly, Bhargava et al. [220] discovered reduced bile acids in patients with MS and the supplementation of bile acid could prevent astrocytic polarization into neurotoxic phenotypes, ameliorating neuropathology in MS models. More importantly, proteomic studies of cerebrospinal fluid from MS patients have highlighted heparin‐binding EGF‐like growth factor (HB‐EGF) as a central factor in tissue protection and anti‐inflammatory responses. It is upregulated in both cortical and spinal cord astrocytes during peak disease stages but declines during later phases. Notably, HB‐EGF is predominantly produced by a proliferative, cortical‐origin astrocyte subset. HB‐EGF is essential for resolving acute inflammatory lesions in autoimmune CNS disorders, mediating protective and inflammatory roles under hypoxic conditions via HIF1α signaling pathways. Therapy with intranasal HB‐EGF could attenuate disease severity in preclinical mouse models of MS [221].

### 4.3 Astrocyte–Oligodendrocyte Interactions and Myelin Integrity

Astrocytes influence myelin integrity and oligodendrocyte function through multiple molecular pathways. The neurotrophin receptor TrkB in astrocytes promotes demyelination in MS. Its upregulation marks chronic demyelinated areas and is associated with the loss of neurotrophic support. TrkB activation induces the expression of the copper transporter CTR1, which enhances copper uptake and release, ultimately causing oligodendrocyte loss [215]. Research indicates the expression of clusterin (CLU) levels is upregulated in astrocytes from MS patient tissues. CLU enhances astrocyte proliferation but hinders myelin debris clearance by astrocytes. From a mechanistic perspective, CLU inhibits the PI3K–AKT pathway in primary OPCs through very‐low‐density lipoprotein receptors, leading to OPC and myelin loss in the corpus callosum. Pharmacological activation of AKT mitigates CLU‐induced OPC damage and alleviates demyelination in the corpus callosum. Furthermore, astrocyte‐specific conditional knockout of CLU in a mouse MS model enhances myelin regeneration and reduces disease severity [212]. The interaction between astrocytes and mature oligodendrocytes is crucial for remyelination. Studies suggest astrocytes promote regenerating oligodendrocyte survival by regulating the Nrf2 pathway associated with increased cholesterol biosynthesis in astrocytes. In male mice with localized CNS injury, sustained activation of astrocytic Nrf2 impairs remyelination but can be restored by stimulating cholesterol biosynthesis/export or inhibiting Nrf2. This research emphasizes the significance of interactions between astrocytes and oligodendrocyte in regulating remyelination and proposes a CNS regenerative therapeutic strategy targeting this interaction [223].

### 4.4 Astrocytes in Neuronal Survival and Cognitive Dysfunction

Astrocytes produce factors that support neuronal survival and regeneration under certain conditions. Kerkering et al. [222] discovered that astrocytes derived from benign MS (BMS) patients could suppress TNF‐α/IL‐17A‐induced neuronal damage. Upon inflammatory stimulation, BMS astrocytes activate the JAK/STAT pathway, leading to the expression of neurotrophic factors such as LIF and TGF‐β1, which play key roles in promoting axonal protection and regeneration and maintaining neuronal homeostasis [222]. Chitinase‐3‐like (CHI3L1), a protein secreted by activated astrocytes, is significantly elevated in MS patients. During hippocampal demyelination, CHI3L1 impairs neurogenesis and cognitive function by disrupting β‐catenin signaling, thereby inhibiting neural stem cell proliferation and differentiation and compromising dendritic development, complexity, and synaptogenesis of neurons. Inhibition of CHI3L1 or restoration of β‐catenin signaling alleviates neuronal damage, suggesting a potential therapeutic target for neurological dysfunction in MS [213].

Astrocytes exhibit dual roles in MS, acting as both protectors and exacerbators of disease. Their effects depend on the molecular pathways they engage. Focusing on astrocyte‐specific signaling pathways offers potential for developing therapeutic approaches in MS.

## 5 Psychiatric Disorder

Psychiatric disorders are caused by pain or damage to vital functional areas. The WHO analysis report shows that psychiatric disorders are prevalent worldwide, with anxiety and depression being the most common [232]. There is an urgent need to study the mechanisms of various psychiatric disorders and to develop drugs. Astrocytes, which are the most numerous glial cells in the CNS, coevolve with neurons over time [233]. Both astrocytes and neurons are deeply involved in the occurrence and development of mental disorders [233], mainly involving abnormalities in the central amygdala, striatum (STR), hippocampus, frontal cortex, and so on. When the brain is stimulated by inflammation, astrocytes are activated and produce a series of responses [234].

Dopamine and serotonin (5‐HT) neurons regulate dynamic effects [235], and many drugs targeting the treatment of mental illness revolve around 5‐HT or dopamine [236], which is enough to show the importance of these two molecules. Astrocytes play an important role in the nervous system, and it is particularly important to emphasize that they can regulate synaptic signaling and inflammatory response. Therefore, the impact of astrocytes on these mental illnesses cannot be ignored.

Changes in 5‐HT content can affect the occurrence of mental illness. 5‐HT positively affects calcium homeostasis in astrocytes and increases neuronal excitability [237]. González‐Arias et al. [238] demonstrated that the reduction of 5‐HT might affect Ca^2+^ signaling in astrocytes.

Changes in dopamine content can also affect the occurrence of mental illness. Petrelli et al. [239] found that astrocytes in the prefrontal cortex expressed vesicle monoamine transporter 2 (VMAT2), organic cation transporter 3 (OCT3), and monoamine oxidase B (MAO‐B), which together worked to maintain dopamine homeostasis. Among them, the loss of VMAT2 led to decrease in extracellular dopamine levels in the prefrontal cortex, enhancement of excitatory synaptic transmission, impairment of synaptic plasticity, and changes in dendritic spines morphology and density. These changes can lead to deficiencies in working memory and cognitive flexibility [239]. Nisha Aji et al. [240] also found that in clinical populations with early psychiatric (FEP) and its high‐risk state (CHR), the levels of MAO‐B were significantly reduced in subcortical regions (especially striatals). This shows that astrocytes may cause their ability to metabolize monoamines such as dopamine, which in turn causes abnormal increase in dopamine levels in areas such as STR, which is consistent with the phenomenon of increased STR dopamine levels observed in psychiatric diseases [240].

**FIGURE 3:**
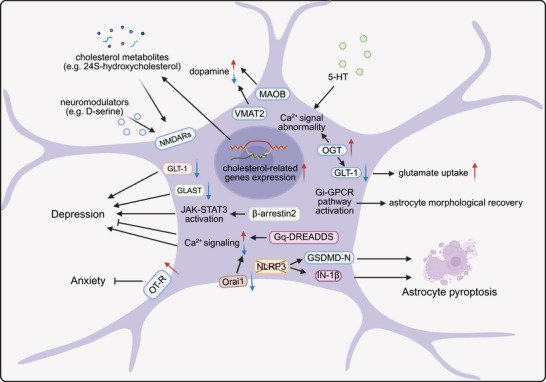
Relevant mechanisms involved in astrocytes in psychiatric disorders. The Ca^2+^ signaling, JAK–STAT3, and Gi–GPCR pathways are mainly involved. Key molecules such as VMAT2, MAOB, NMDARs, GLT‐1, GLAST, and so on, involved in related signaling pathways, can alleviate or aggravate the symptoms of related psychiatric disorders. In addition, pathways associated with glutamate uptake, astrocyte morphological restoration, and astrocyte pyroptosis are also shown. The figure was created using BioRender.com.

In psychiatric disorders, glutamate excitotoxicity and abnormal Ca^2+^ signaling pathway have also attracted the attention of many researchers. The main pathways and key molecules of astrocytes in different mental disorders are described below (Table 6 and Figure 3).

**TABLE 6:** Molecules and mechanisms associated with astrocytes in psychiatric disorder.

Disease	Key molecules/signaling pathways	Implications	References
Depression	Orai1	Orail1 deletion attenuates inflammation as well as the Ca^2+^ signaling pathway to ameliorate depressive‐like behavior.	[245]
	NLRP3/caspase‐1/GSDMD	Decreased expression levels of GSDMD‐N and IL‐1β due to loss of NLRP3 can attenuate CMS‐induced astrocyte pyroptosis and alleviate depressive symptoms.	[246]
	GLAST, GLT‐1	HFD‐induced downregulation of glial glutamate transporters, GLAST, and GLT‐1 led to the observed circuit maladaptation and subsequent depressive‐like behavior.	[243]
	Ca^2+^ signaling pathway	Decreased 5‐HT‐mediated Ca^2+^ signaling in Cort‐mice's mPFC astrocytes results in aberrant 5‐HT‐driven synaptic plasticity in mPFC neurons.	[238]
	β‐arrestin 2	Deletion of β‐arrestin 2 gene downregulates β‐arrestin 2, thereby reducing its binding to STAT3 and activating the JAK‐STAT3 signaling pathway.	[244]
	Sirt1/Nrf2/HO‐1/Gpx4 axis	Edaravone can exert antidepressant and anxiolytic effects by modulating the Sirt1/Nrf2/HO‐1/Gpx4 axis.	[248]
	Glutamate excitotoxicity, Ca^2+^ signaling pathway	After specific knockdown of OGT, GLT‐1 was significantly reduced, resulting in enhanced glutamate uptake and attenuating the damage of glutamate excitotoxicity. Vertebral neuronal calcium activity remained normal in mPFC.	[242]
	5‐HT_2B_R/β‐arrestin2 pathway	Fluoxetine inhibits the activation of reactive A1 astrocytes in a mouse model of MDD via the astrocyte 5‐HT_2B_R/β‐arrestin2 pathway.	[247]
Anxiety	OT	Astrocytes express OT receptors and mediate the anxiolytic and positive strengthening effects of OT in the central amygdala.	[249]
	NMDAR	NMDAR plays an important role in vHPC astrocyte‐mediated anxiety‐like behavior. It can be reversed with NMDAR antagonists.	[250]
		Hippocampal astrocytes are activated by optogenetic techniques to release ATP to increase the frequency of sEPSCs in hippocampal dentate gyrus granulosa cells, thereby regulating synaptic transmission and producing anxiolytic effects.	[251]
OCD	SAPAP3, GLT1	SAPAP3 is involved in glutamate uptake and the formation of the actin cytoskeleton. GLT1 uptakes glutamate from the synaptic cleft back to astrocytes to regulate glutamate concentration and neurotransmission.	[233]
	Gi–GPCR signaling pathway	Activation regulation of the Gi–GPCR signaling pathway in astrocytes can correct the compulsive behaviors that occur in Sapap3 KO mice.	[252]
Schizophrenia	NMDAR	In SNAP, astrocytes specifically express genes related to cholesterol synthesis, fatty acid metabolism, and synaptic adhesion, affecting the function of NMDARs.	[257]
	*DISC1*, D‐serine, NMDA receptor	Astrocytes regulate their excitability by releasing neuromodulators such as D‐serine and acting on NMDARs in *WFS1*‐positive neurons.	[258]
	*Grin2a*	The *Grin2a* mutation activates the astrocyte cholesterol biosynthesis pathway, which is a non‐cell‐autonomous indirect effect.	[256]

Abbreviations: 5‐HT, 5‐hydroxytryptamine; 5‐HT_2B_R, 5‐HT_2B_ receptor; CMS, chronic mild stress; Gi–GPCR, inhibitory G protein‐coupled receptor; GLAST, glutamate–aspartate transporter; GLT1, glutamate transporter 1; GLT‐1, glutamate transporter 1; GSDMD‐N, gasdermin D N‐terminal domain; HFD, high‐fat diet; IL‐1β, interleukin‐1β; MDD, major depressive disorder; mPFC, medial prefrontal cortex; NLRP3, NLR family, pyrin domain containing 3; NMDAR, N‐methyl‐d‐aspartate receptor; OCD, obsessive compulsive disorder; Orail1, orai calcium channel protein 1; OT, oxytocin; SAPAP3, synaptic postsynaptic density‐associated protein 3; sEPSCs, spontaneous excitatory postsynaptic currents; SNAP, the synaptic neuron and astrocyte program; STAT3, signal transducer and activator of transcription 3; vHPC, ventral hippocampus.

### 5.1 Astrocytes in Depression

By affecting the neuronal uptake of glutamate (an excitatory neurotransmitter), astrocytes exert a significant influence on depression [241]. In the CSDS model, O‐GlcNAc transferase (OGT) expression in astrocytes was significantly upregulated in the prefrontal cortex (medial PFC [mPFC]) of susceptible mice. After specific knockdown of OGT, glutamate transporter 1 (GLT‐1) was significantly reduced, resulting in increased glutamate uptake, which insulated neurons from damage by glutamate excitotoxicity. When OGT was knocked out in astrocytes, vertebral neuronal calcium activity remained normal in mPFC [242]. Tsai et al. [243], utilizing lentiviral knockout and overexpression, demonstrated that high‐fat diet (HFD)‐induced downregulation of GLAST and GLT‐1 led to the observed circuit maladaptation and subsequent depressive‐like behavior. On the contrary, riluzole had the ability to ameliorate HFD‐induced behavioral impairments by normalizing the expression levels of GLAST and GLT‐1, as well as the afferent neurons in the ventral hippocampus that projected to the thalamic nucleus [243]. The development of depression is inextricably linked to neuroinflammatory stimuli. Liu et al. [244] found that downregulation of β‐arrestin 2 in astrocytes reduced binding to STAT3, which activated the JAK–STAT 3 signaling pathway, leading to the development of neuroinflammation and a depressive‐like phenotype.

In addition, neuroinflammation‐mediated abnormal Ca^2+^ signaling is an important mechanism leading to depression. Novakovic et al. [245] found that astrocytes lacking Orai1 (calcium signaling channel) released less proinflammatory cytokines when stimulated by peripheral LPS, thereby reducing inflammation levels. Deletion of Orai1 also attenuated inflammation‐induced Ca^2+^ signaling in astrocytes and inhibitory neurotransmission in the hippocampus, which helped attenuate depression‐like behavior [245]. Of course, the Ca^2+^ signal can also be affected by other factors. González‐Arias and colleagues [238] found a decrease in serotonin (5‐HT)‐mediated Ca^2+^ signaling and aberrant 5‐HT‐driven synaptic plasticity in mPFC astrocytes of corticosterone‐treated juvenile mice (Cort‐mice) and abnormal 5‐HT‐driven synaptic plasticity in 2/3 layers of mPFC neurons. They also found that Gq‐DREADDS could enhance Ca^2+^ signaling in astrocytes, thereby restoring Cort‐mice's mood and cognitive abilities to control levels [238].

In addition to the possible mechanisms common to the two psychiatric disorders mentioned above, depression can also be affected by astrocyte pyroptosis, abnormal activation, and so on. Li et al. [246] validated the effect of astrocyte pyroptosis in depression with an antidepressant, a selective serotonin reuptake inhibitor. They also demonstrated that NLRP3 deletion downregulated the expression levels of GSDMD‐N and IL‐1β, and attenuated chronic mild stress (CMS)‐induced astrocyte pyroptosis and alleviated depressive symptoms [246]. Fang et al. [247] validated that fluoxetine inhibited the activation of reactive A1 astrocytes in a mouse model of MDD via the 5‐HT_2B_ R/β‐arrestin2 pathway.

### 5.2 Astrocytes in Anxiety Disorder

It is possible that depression is strongly associated with other neuropsychiatric disorders because the effects of changes in astrocyte morphology or function are not confined to one disease [241]. It is suggested that in the CSDS model, neuroinflammation is induced, astrocytes in the hippocampus and prefrontal cortex are dysfunctional, reducing the production of antioxidants and exacerbating oxidative stress damage. Dang et al. [248] found that edaravone could exert antidepressant and anxiolytic effects by modulating the Sirt1/Nrf2/HO‐1/Gpx4 axis, in which Gpx4‐mediated ferroptosis might play a key role.

The mechanism of astrocytes in anxiety disorder is very similar to that of depression, affecting the flow of glutamate excitatory neurons and calcium ions, but the specific sites of action are slightly different. Wahis et al. [249] found that astrocytes could also express OT receptors and mediate the anxiolytic and positive enhancement effects of OT in the central amygdala of mice and rats. Li et al. [250] demonstrated increased vHPC astrocyte activity in the anxious environment and 3‐d subacute restraint stress, a valid mouse model of anxiety disorder, leading to increased glutamate levels and neuronal dysfunction. Application of NMDARs antagonists could reverse anxiety symptoms caused by activation of vHPC astrocytes [250]. Cho and colleagues [251] found that calcium activity in astrocytes might reflect anxiety in mice. They activated hippocampal astrocytes through optogenetic techniques to release ATP, which in turn increased the frequency of excitatory postsynaptic currents in hippocampal dentate gyrus granulosa cells, thereby regulating synaptic transmission and producing anxiolytic effects [251].

### 5.3 Astrocytes in Obsessive Compulsive Disorder

Similarly, glutamate excitotoxicity plays an important role in obsessive compulsive disorder (OCD). Soto and colleagues [233] demonstrated that GLT1 in astrocytes was responsible for the uptake of glutamate from the synaptic cleft back into astrocytes. This process regulated glutamate concentration and neurotransmission. Dysfunctional GLT1 might affect glutamate‐mediated neurotransmission, which in turn was associated with the pathogenesis of OCD [233]. In addition, a team led by Joselyn S. Soto found that astrocyte morphology was impaired in Sapap3 KO mice. Activation of the Gi–GPCR signaling pathway could restore the morphology of astrocytes by regulating the homeostasis of the actin cytoskeleton and improve their function by restoring the expression of transporters and channels. At the same time, it could also significantly reduce the excitability of mesospinous neurons. Activation of the Gi–GPCR signaling pathway in astrocytes ultimately manifested itself in the correction of compulsive behaviors in Sapap3 KO mice [252].

### 5.4 Astrocytes in Schizophrenia

Single‐cell sequencing studies related to schizophrenia are beginning to bear fruit. Szabo et al. [253] found an enrichment of many SCZ‐related DEGs in astrocytes by sequencing, especially in the MFC, STR, and temporal lobe regions. They went on to explore the functional effects and found that downregulation of these DEGs led to abnormal calcium signaling, decreased glutamate uptake, and decreased MMPs activity in astrocytes [253].

Methylation of related genes may be strongly associated with the development of schizophrenia. The team led by Abdolmaleky et al. [254] found that the expression of genes associated with astrocyte differentiation, such as *SOX9* and *NR2E1*, was upregulated in patients with schizophrenia. Among them, the increase in gene expression is due to the hypomethylation of its promoter. In addition, the expression of TGFB2, a key ligand in the TGFβ signaling pathway, was also significantly increased in astrocytes [254]. Chan et al. [255] found that methylation of *ALDH1A2* could affect the content of retinoic acid, thereby affecting the occurrence of schizophrenia.

In schizophrenia, dysfunction of astrocytes may result in abnormal synaptic pruning, inadequate neuronal support, and increased release of inflammatory mediators, resulting in structural changes in the brain. Current research is more focused on abnormalities in the cholesterol synthesis pathway in astrocytes. There is a study through transcriptome analysis revealing that astrocytes in *Grin2a* mutant mice were significantly altered, especially the expression of genes related to cholesterol biosynthesis pathway was upregulated. While *Grin2a* was mainly expressed in neurons, the mRNA levels of *Grin2a* in astrocytes were low, reflecting the indirect effects of noncellular autonomy [256]. Ling's team [257] found that in the synaptic neuron and astrocyte program, astrocytes specifically expressed genes related to cholesterol synthesis, fatty acid metabolism, and synaptic adhesion. Cholesterol was not only a key component of the synaptic membrane, but also affected the function of NMDARs through its metabolites such as 24S‐hydroxycholesterol [257]. Zhou et al. [258] found that astrocytes regulated the excitability of WFS1‐positive neurons by releasing neuromodulators such as D‐serine to act on NMDARs in WFS1‐positive neurons.

## 6 Discussion

**FIGURE 4:**
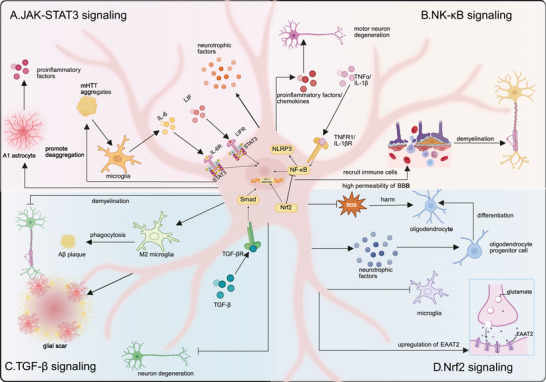
Four common pathways for neuropsychiatric disorders associated with astrocytes. This figure illustrates the involvement of four key signaling pathways: (A) JAK–STAT3, (B) NF–κB, (C) TGF‐β, and (D) Nrf2 in astrocytes, involving their interactions with other neural and immune cell types in the pathophysiology of neuropsychiatric disorders. (A) *JAK–STAT3 signaling*: Astrocyte activation of the JAK–STAT3 pathway contributes to the formation of A1 neurotoxic astrocytes, thereby exacerbating neuronal damage and promoting motor neuron degeneration. STAT3 activation also promotes microglia‐mediated depolymerization of mutant huntingtin protein (mHTT) aggregates. (B) *NF‐κB signaling*: Activation of NF‐κB in astrocytes leads to an exacerbation of neuroinflammation and even demyelination and neuronal damage by causing motor neuron degeneration and BBB dysfunction. (C) *TGF‐β signaling*: TGF‐β signaling limits neuroinflammation. Its chronic activation contributes to glial scarring and inhibits demyelination and neuronal degeneration. (D) *Nrf2 signaling*: Activation of the antioxidant transcription factor Nrf2 in astrocytes leads to upregulation of neuroprotective genes, increasing neurotrophic factor production, enabling oligodendrocyte differentiation, and attenuating ROS‐induced cell damage, thereby exerting a protective effect on neurodegeneration. The diagram was created using BioRender.com.

Astrocytes are critical regulators in the CNS, contributing to various physiological and pathological processes, particularly in CNS diseases [1]. Previous studies have emphasized their dual role as both protective and detrimental players, highlighting opportunities for targeting astrocyte‐specific mechanisms in therapeutic interventions [259]. In the previous studies on neurological diseases, multiple key targets showed cross‐disease importance. Among them, Nrf2 is a core target that co‐occurs in all three diseases (MS, AD, and psychiatric disorders), while molecules such as IL‐3 (MS, AD), TGF‐β1 (MS, ALS), C1q (AD, ALS), CHI3L1 (MS, AD), and NMDAR (psychiatric disorders, HD) play a role in both diseases. The regulatory networks of the four major signaling pathways commonly involved in these neurological disorders within astrocytes are depicted in Figure 4. These targets mainly regulate key pathological processes such as oxidative stress, neuroinflammation, and synaptic function, suggesting that interventions targeting these common pathways may offer broad‐spectrum therapeutic prospects for a variety of neurological diseases. Future studies need to further elucidate the commonality and specificity of these targets across diseases to develop more precise cross‐disease therapeutic strategies.

However, the traditional binary classification of astrocytes into A1 and A2 phenotypes, presents notable limitations in fully capturing the complexity of the regulation of astrocytes in CNS, ignoring their context‐dependent responses and the influence of microenvironmental factors. Current reviews on CNS diseases and astrocytes often lack a comprehensive discussion of astrocyte heterogeneity without adequately considering the complex and dynamic responses of astrocyte subtypes in distinct pathological contexts. Our review addresses this critical gap by providing a detailed exploration of astrocyte heterogeneity in a range of CNS diseases, emphasizing the distinct transcriptional and functional profiles of astrocytes, in order to offer unique insights into the complexity of astrocyte‐mediated pathology and their therapeutic potential.

A significant strength of existing research lies in the identification of astrocyte subtypes and molecular pathways involved in disease progression [260]. For example, studies have revealed the contributions of astrocytes to neuroinflammation, oxidative stress, and synaptic regulation through mechanisms such as glutamate excitotoxicity and calcium signaling [2, 41, 261, 262]. These findings underscore the role of astrocytes in neurodegenerative diseases such as AD, PD, and MS. Notably, recent advancements in single‐cell sequencing have provided transformative insights into astrocyte heterogeneity, enabling a more precise characterization of their roles in various neurologic conditions [5]. An increasing number of studies have focused on the interactions between astrocytes and other cell types, including neurons, microglia, and oligodendrocyte, which is essential for understanding of astrocyte‐mediated regulation in maintaining homeostasis, their dysregulation in pathological conditions, and the identification of novel therapeutic targets [263].

Despite these achievements, several challenges persist. One major limitation is the difficulty in linking transcriptionally characterized astrocyte subtypes to their real‐time functions, neuronal dynamics, and disease‐specific features. Moreover, while numerous astrocyte‐targeted molecules and signaling pathways have been identified, translating these findings into effective clinical interventions is still in its infancy. The investigation into the crosstalk between astrocytes and other cell types remains insufficiently comprehensive, further complicating the development of comprehensive treatment strategies.

Additionally, many studies have focused on individual diseases, limiting the exploration of the shared mechanisms of astrocytes across various conditions. The interplay between astrocyte functions in neurodegeneration and psychiatric disorders, such as JAK‐STAT 3 signaling in MS and depression [222, 244], suggests overlapping pathways that could yield broader therapeutic implications if investigated more thoroughly.

Future research should prioritize the integration of advanced technologies, such as single‐cell RNA sequencing, spatial transcriptomics and high‐resolution imaging to better capture astrocytic dynamics and heterogeneity in human disease progression and resolution [264]. Investigating the molecular drivers of RA sub‐states, as defined by transcriptomic profiles, is critical for elucidating their functional changes in different pathological contexts [5]. The development of astrocyte‐targeted therapies represents a promising frontier in CNS disease treatment. Strategies such as gene editing, metabolic modulation, and subtype‐specific interventions hold significant potential. Several small molecules with CNS penetrance have been validated for efficacy in animal models [175, 182, 201]. Moreover, neural stem/progenitor cell‐derived extracellular vesicles demonstrate unique benefits, such as crossing the BBB, low immunogenicity, and the ability to deliver neuroprotective and immunomodulatory components. Their application has been shown to mitigate behavioral and pathological symptoms in both preclinical and clinical models [265].

Currently, a growing body of clinical trials has begun to focus on the critical role of astrocytes in the progression of neurological diseases, reflecting significant advancements in this field (Table 7). However, astrocytes exhibit marked regional and functional heterogeneity within the CNS, complicating the extrapolation of preclinical findings to human patients. Commonly used animal models, particularly rodent systems, fail to fully recapitulate the molecular and physiological characteristics of human astrocytes, thereby limiting the translational relevance of experimental results. The functional complexity of astrocytes further adds to the challenge, as therapeutic modulation of these cells may elicit unintended consequences. This underscores the urgent need for a comprehensive understanding of astrocyte roles across various neurological disorders and the development of precise, cell‐specific interventions. Moreover, the current lack of molecular agents capable of selectively targeting specific astrocyte subtypes or functional states hinders the advancement of precision therapies. Effective delivery of therapeutics across BBB to astrocytes remains another unresolved barrier, raising concerns about bioavailability and off‐target effects. In addition, the absence of robust, astrocyte‐specific biomarkers impedes dynamic monitoring of astrocyte activity and therapeutic responses. Addressing these gaps is essential for advancing astrocyte‐focused strategies from bench to bedside.

**TABLE 7:** Clinical trials associated with astrocytes in neurological diseases.

Disease	Experimental drugs	Effect	Clinical trial registration number
IS	Glibenclamide	There was no significant difference in the reduction of cerebral edema compared with no glibenclamide.	NCT02864953
	Tirofiban	In patients with acute IS due to occlusion of large vessels in the anterior circulation, intravenous tirofiban prior to endovascular thrombectomy does not reduce disability for 90 days.	ChiCTR‐IOR‐17014167
	MicroRNA‐494 (Observational)	Unknown	NCT03577093
Hemorrhagic stroke	S100B(Observational)	Recruiting	NCT04795362
AD	[18F] RP‐115	Recruiting	NCT05374278
	[11C] BU99008	Terminated (Patient recruitment issues)	NCT02874820
	Mecobiline hydrochloride	Recruiting	ChiCTR1800016198
Psychiatric disorder	Esketamine	Esketamine in combination with ECT can significantly improve depressive symptoms.	ChiCTR2000033118
PD	Simvastatin	This phase II trial is needed to inform the decision to progress to an eventual phase III randomized controlled trial to evaluate the efficacy of simvastatin as a neuroprotective agent in the treatment of PD.	NCT02787590
	NeuroEPO	There was no significant difference between the two groups in terms of the incidence of adverse events, which in both groups were minor, transient, did not require treatment, and had no sequelae.	NCT04110678
	Minocycline	Unknown	NCT00063193
HD	Riluzole	Unknown	NCT00277602
	EGCG	Unknown	NCT01357681
ALS	Astrocyte (AstroRx) cells	A single dose of AstroRx was safe and well tolerated, with signs of clinical improvement observed in the first 3 months after injection.	NCT03482050
	Thalidomide	The frequent and severe THL‐induced bradycardia may stem from subclinical autonomic nervous system involvement in ALS, discouraging its further clinical use.	NCT00231140
	CNS10–NPC–GDNF	A single transplantation of engineered neural progenitor cells can supply new support cells and deliver GDNF to the spinal cord of ALS patients for as long as 42 months following the procedure.	NCT02943850

Research into astrocyte heterogeneity offers a critical theoretical foundation and technical roadmap for the development of precision therapies in CNS diseases. Single‐cell technologies have enabled the identification of disease‐specific or region‐specific astrocyte subtypes and associated signaling pathways, thereby facilitating the design of targeted interventions that minimize disruption to the physiological functions of normal astrocytes. This approach holds promise for enhancing both the safety and efficacy of astrocyte‐directed treatments. Moreover, characterizing astrocyte heterogeneity contributes to the discovery of highly sensitive and specific biomarkers, which are essential for early diagnosis, disease progression monitoring, and therapeutic response evaluation. Looking forward, regenerative strategies that involve the differentiation of induced pluripotent stem cells or in vivo reprogramming into functionally tailored astrocyte subtypes may enable precise cellular replacement in affected brain regions. By leveraging the heterogeneity of astrocytes, researchers can uncover novel therapeutic targets and devise strategies to mitigate the burden of CNS diseases. Given their central role in CNS homeostasis and pathology, astrocytes remain a promising yet underutilized target for advancing the understanding and treatment of CNS diseases.

## Author Contributions

Shijie Mao, Rui Qiao, Qi Wang, and Ling Shen conceptualized the structure of the manuscript, conducted the literature review, and drafted the first manuscript. They worked together to conceptualize the figures and tables, with Shijie Mao and Qi Wang drawing the figures, Shen Ling suggesting and guiding the modifications to the pictures, and Shijie Mao, Rui Qiao, and Qi Wang working together to create the tables. Daxing Li, Xinchen Huo, Jindou Wang, Kunxuan Liu, Wenjing Chen, Tianhao Zhu, Beicheng Zhang, and Shuo Leng assisted in reviewing the manuscript and provided critical feedback. Ying Bai and Shuo Leng were the corresponding author and were responsible for the guidance of the whole project. All authors have read and approved the final manuscript.

## Ethics Statement

The authors have nothing to report.

## Conflicts of Interest

The authors declare no conflicts of interest.

## Data Availability

The authors have nothing to report.
